# Supplementary material for: The Combined Effects of the Most Important Dietary Patterns on the Incidence and Prevalence of Chronic Renal Failure: Results from the US National Health and Nutrition Examination Survey and Mendelian Analyses
Source: Nutrients. 2024 Jul 12;16(14):2248. doi: 10.3390/nu16142248 (PMC11280344; doi:10.3390/nu16142248)
Supplement: Supplementary file 1 [file nutrients-16-02248-s001.zip › Figure S15.pdf]

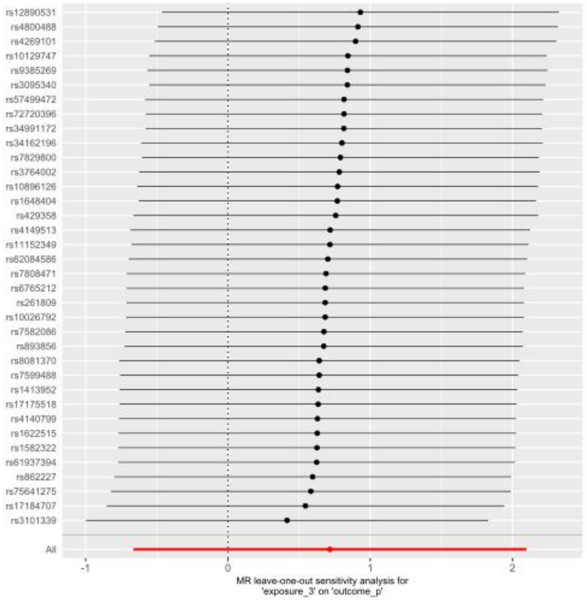

Dried fruit intake

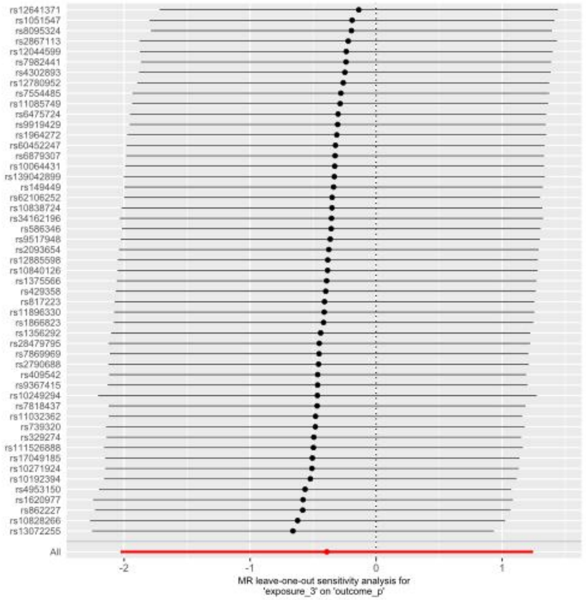

Fresh fruit intake

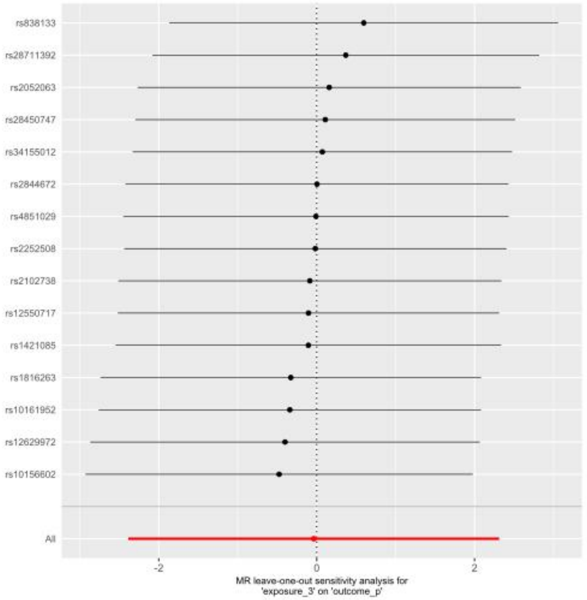

Cooked vegetable intake

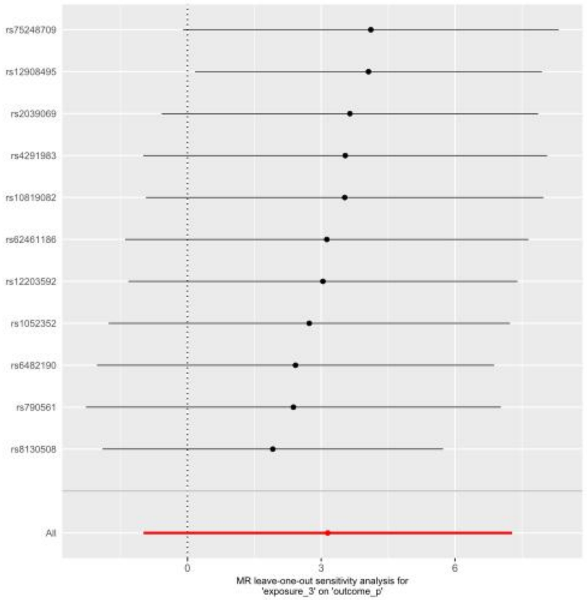

Salad / raw vegetable intake

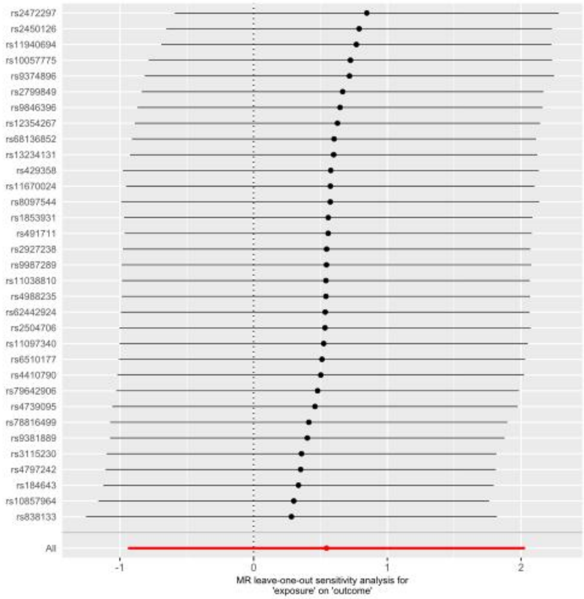

Cereal intake

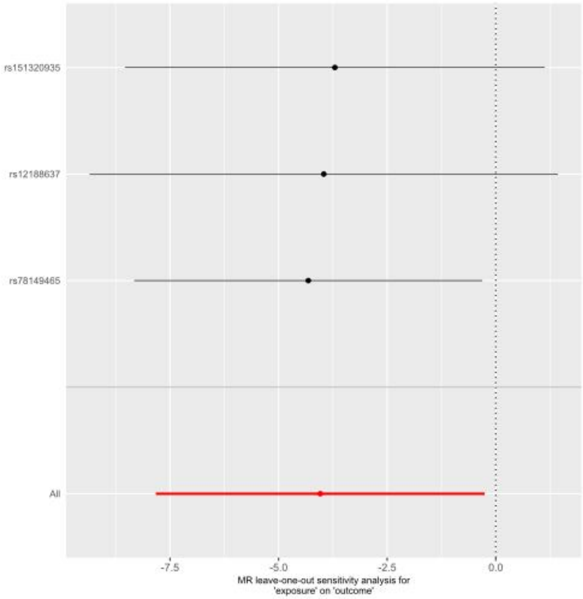

White rice intake

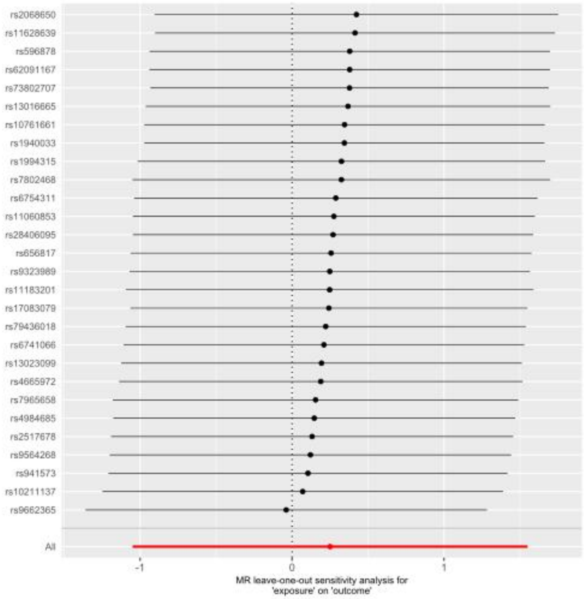

Bread intake

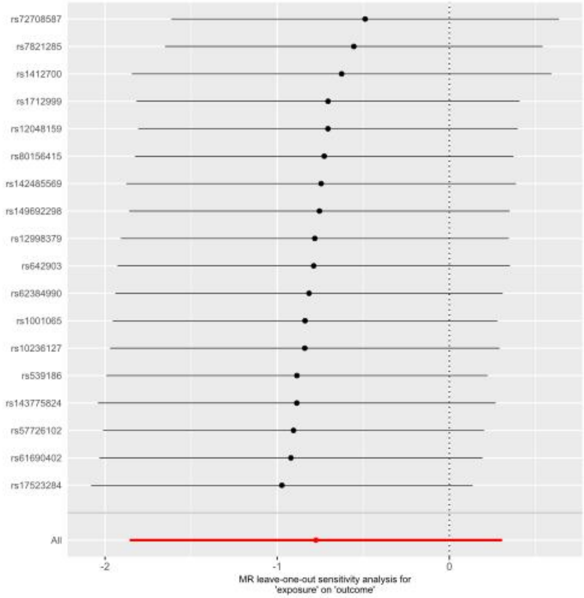

Whole-wheat cereal intake

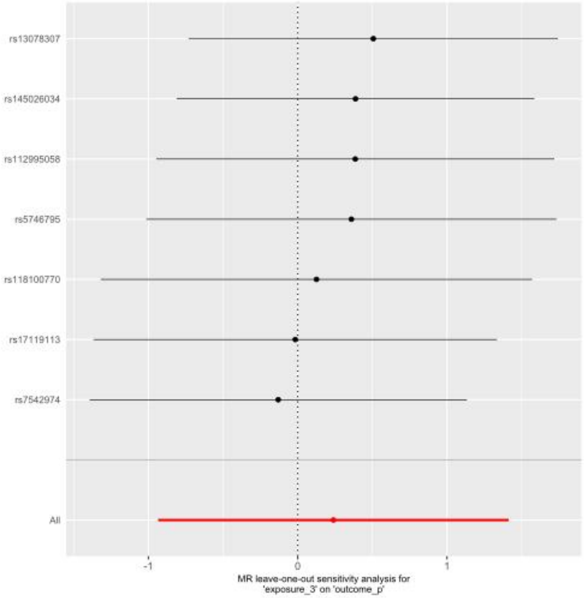

Englyst dietary fibre

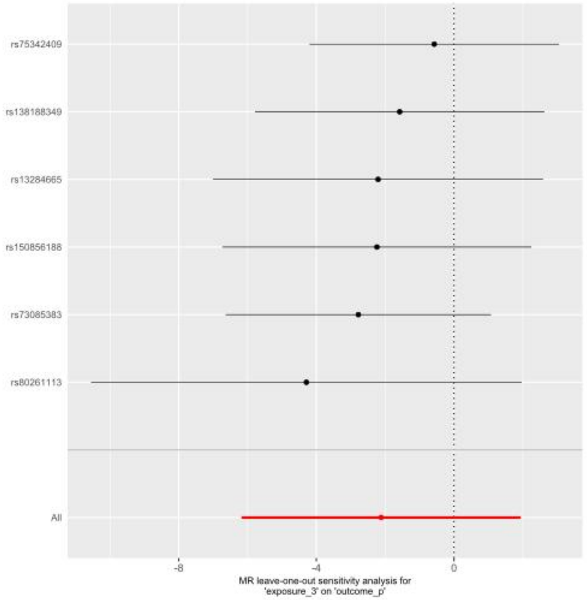

Salted nuts intake

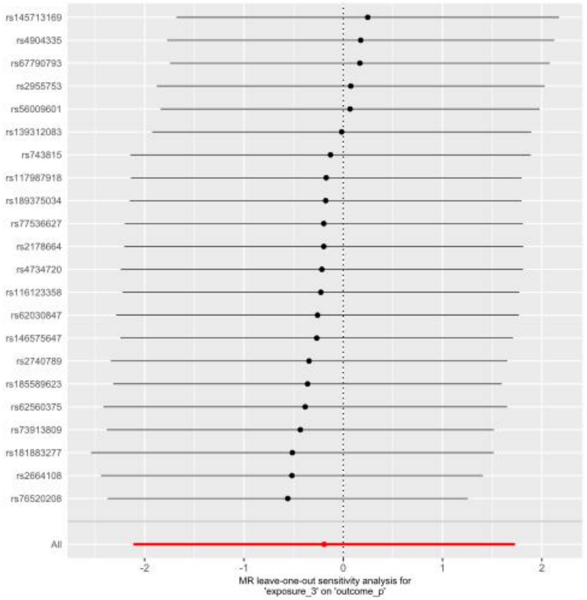

Unsalted nuts intake

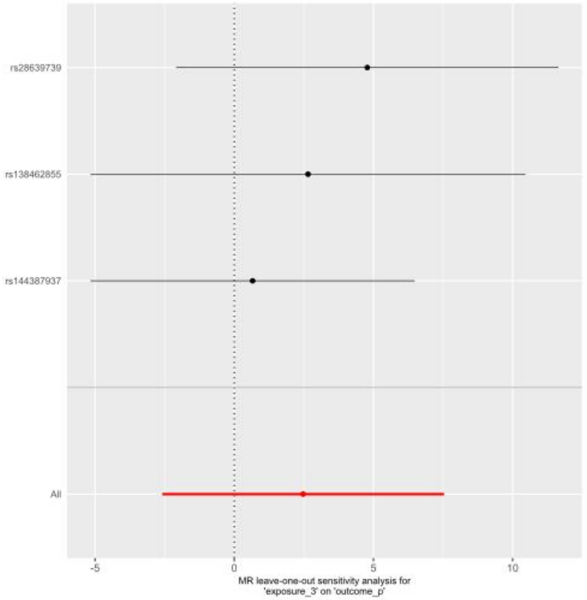

Salted peanuts intake

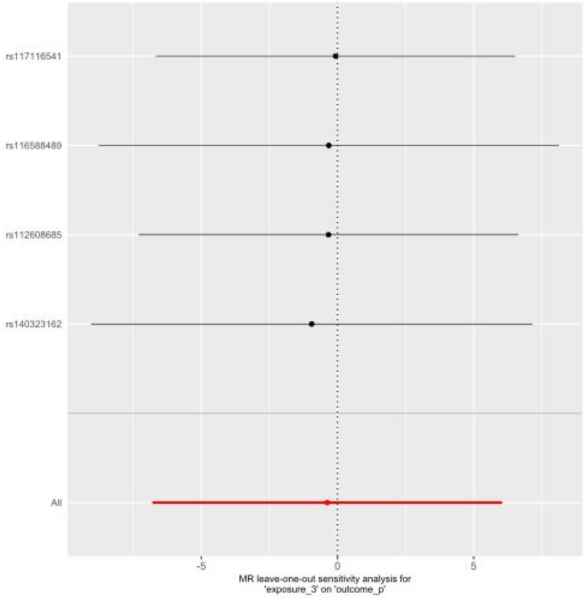

Unsalted peanuts intake

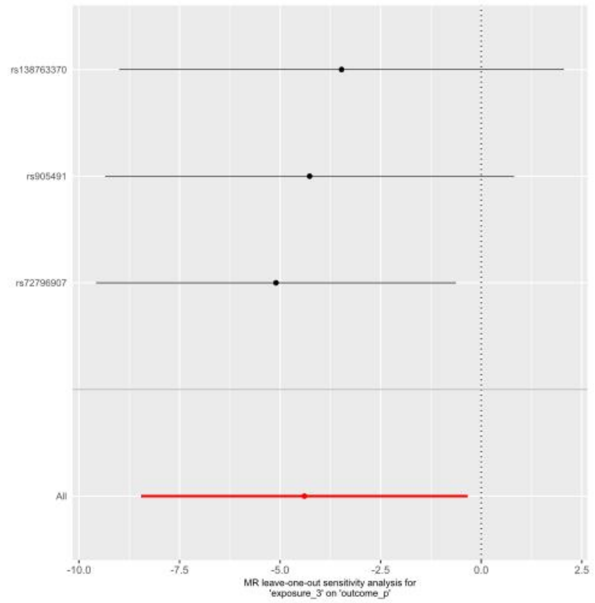

Broad bean intake

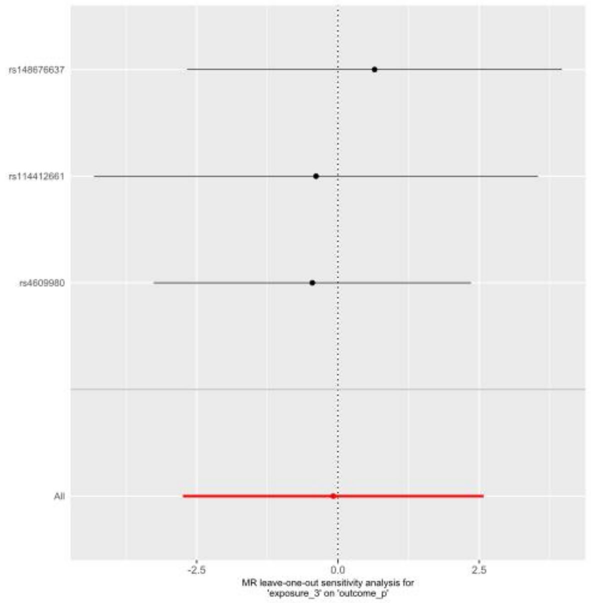

Baked bean intake

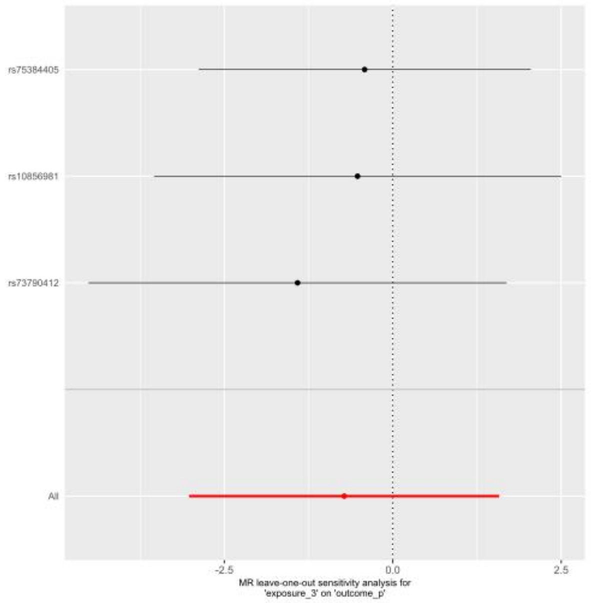

Green bean intake

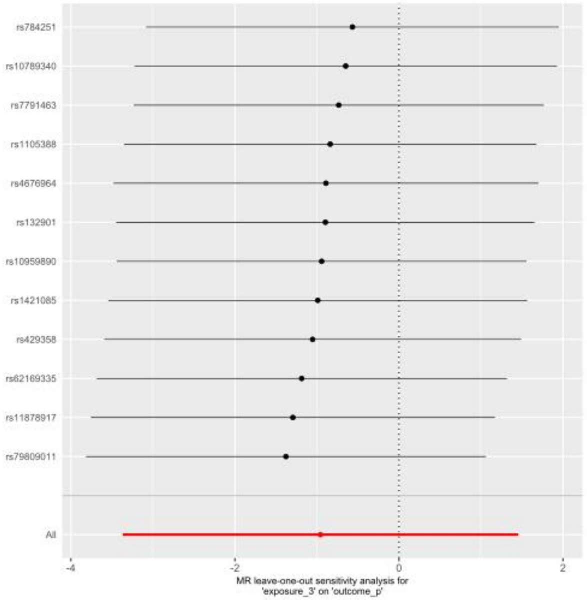

Beef intake

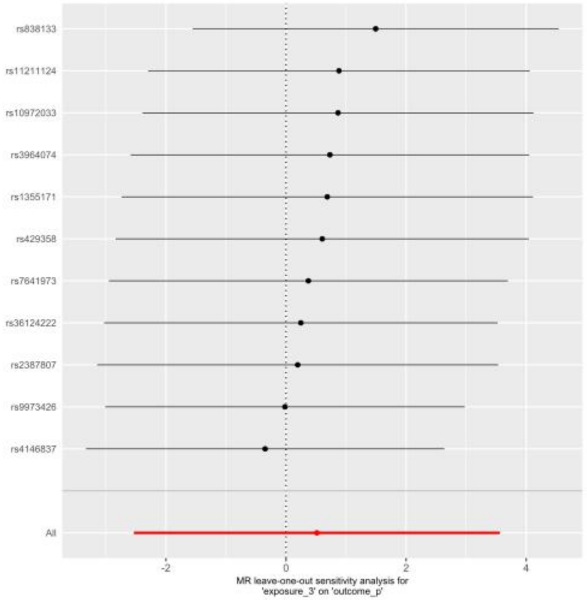

Pork intake

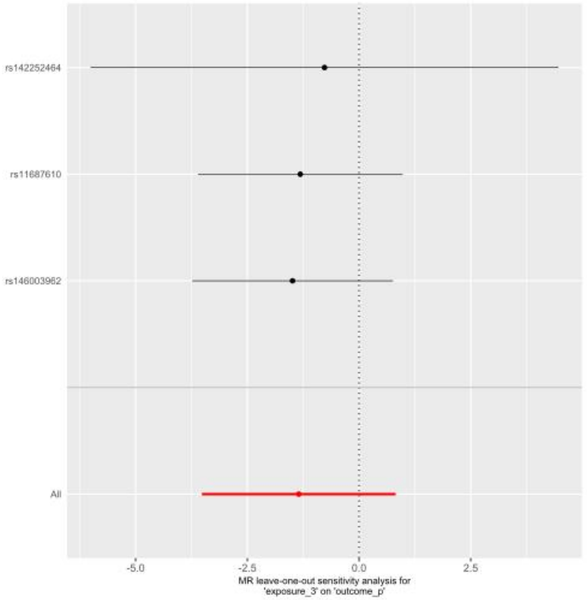

Lamb intake

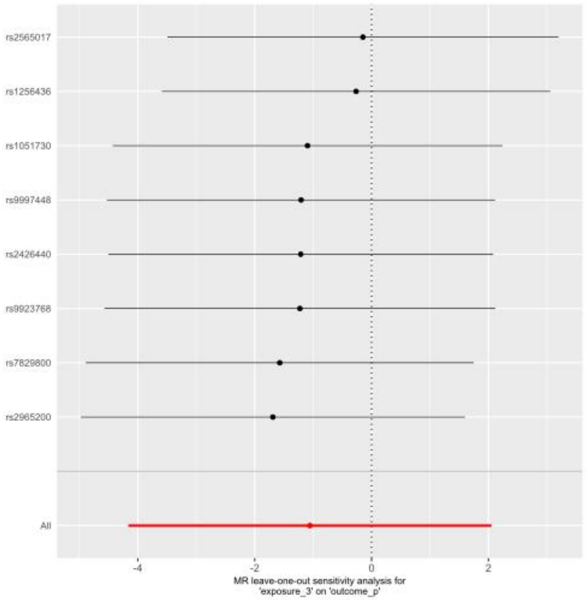

Poultry intake

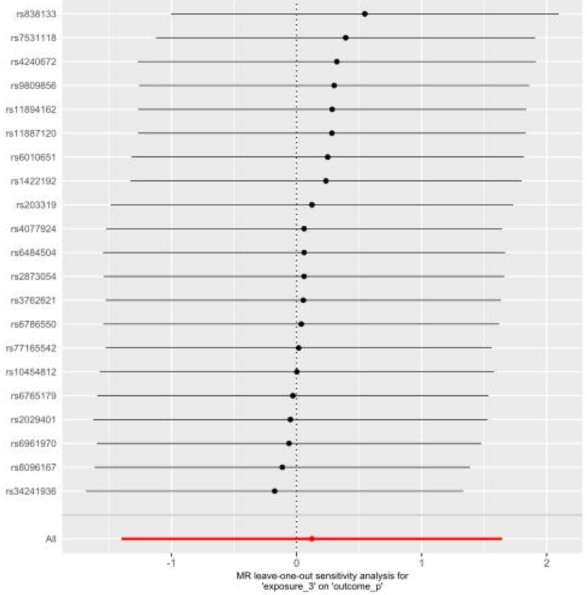

Processed meat intake

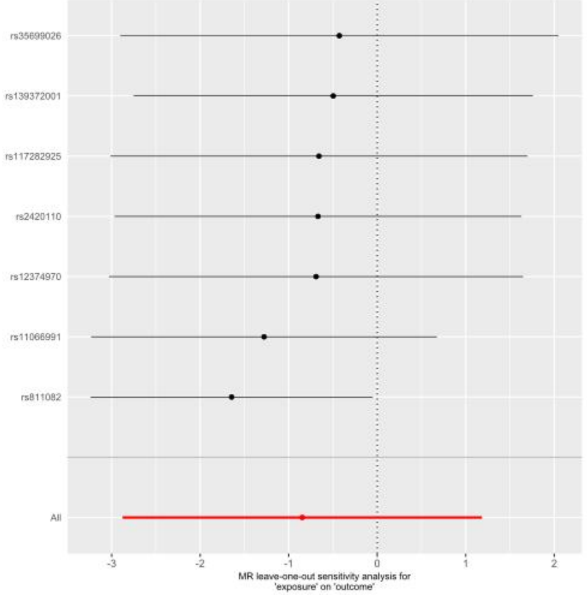

Whole egg intake

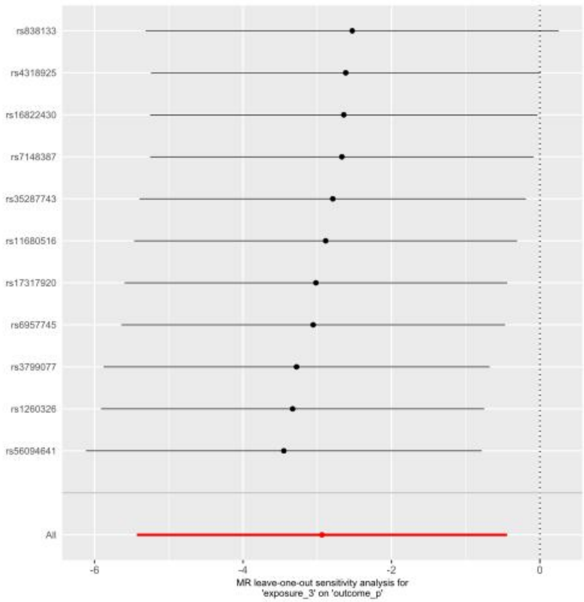

Non-oily fish intake

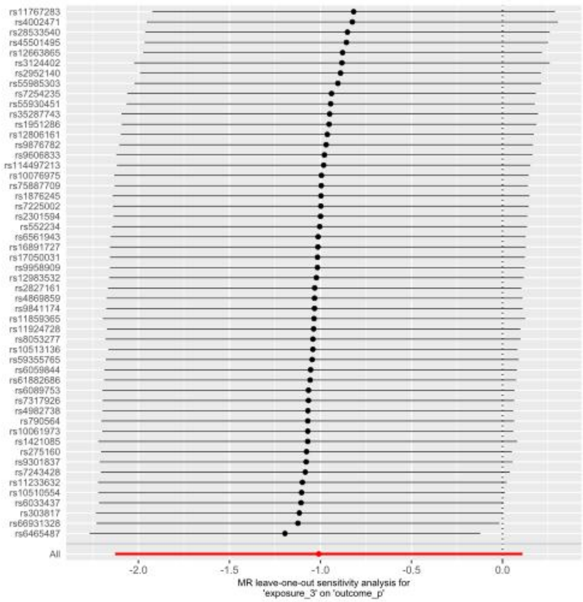

Oily fish intake

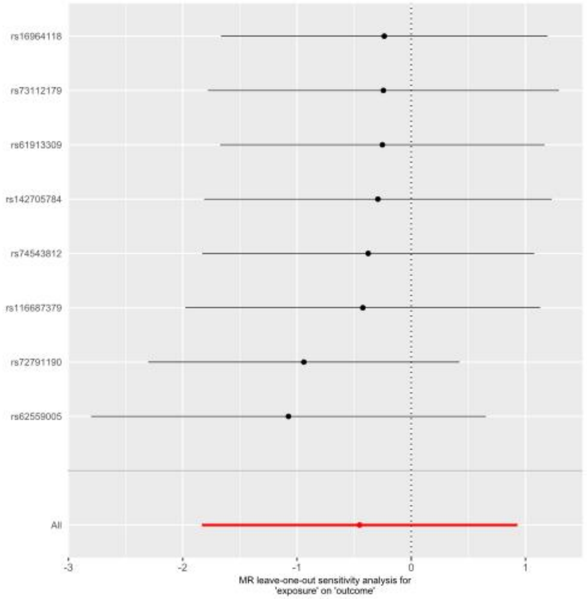

Sweets intake

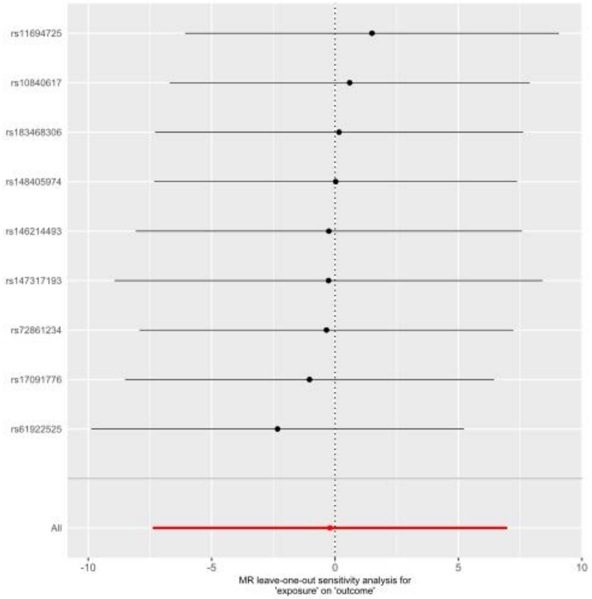

Soya dessert intake

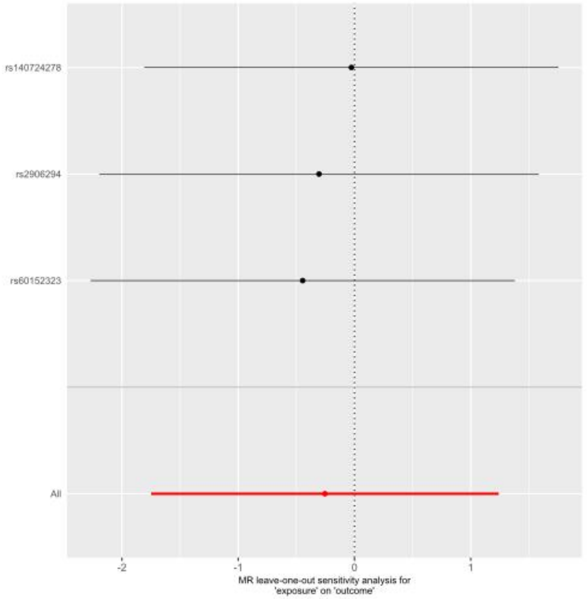

Fried potatoes intake

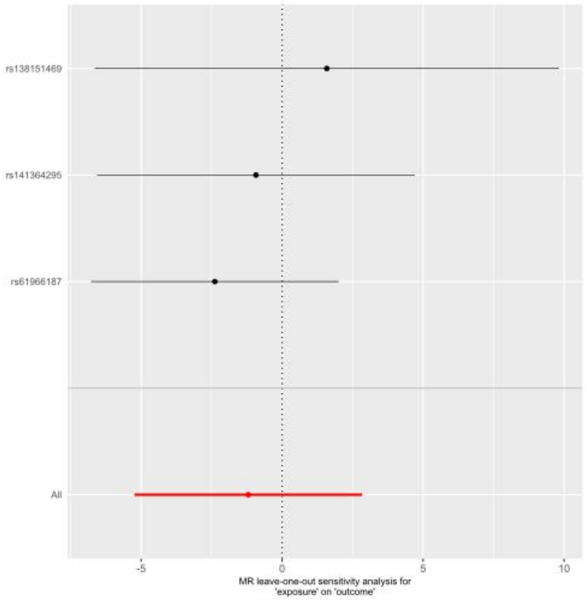

Indian snacks intake

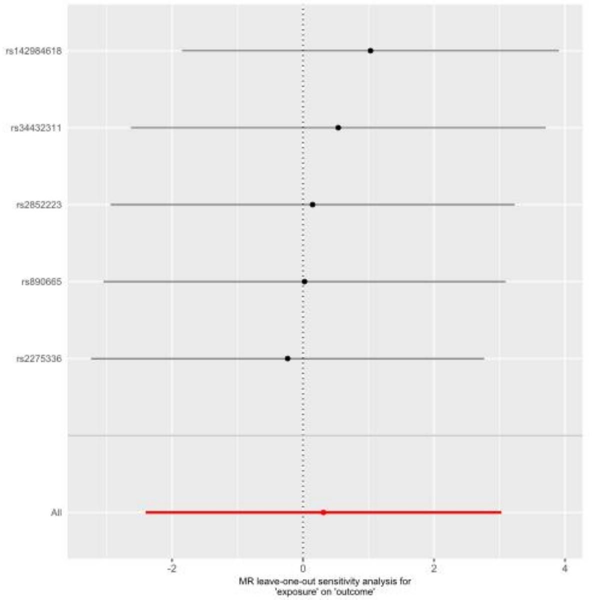

Other dessert intake

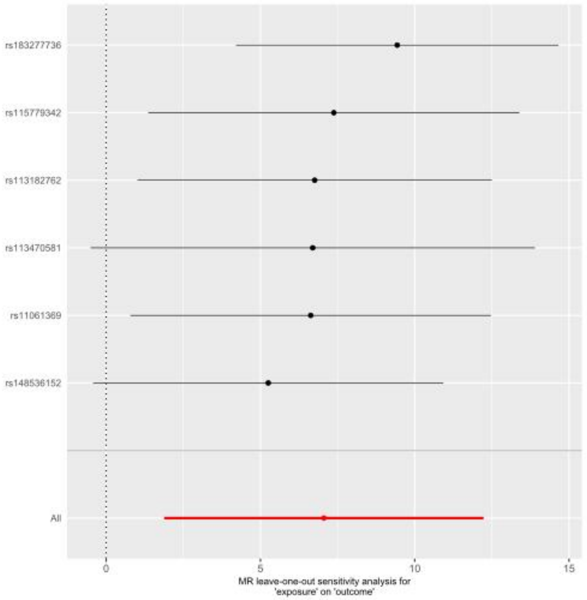

Other savoury snack intake

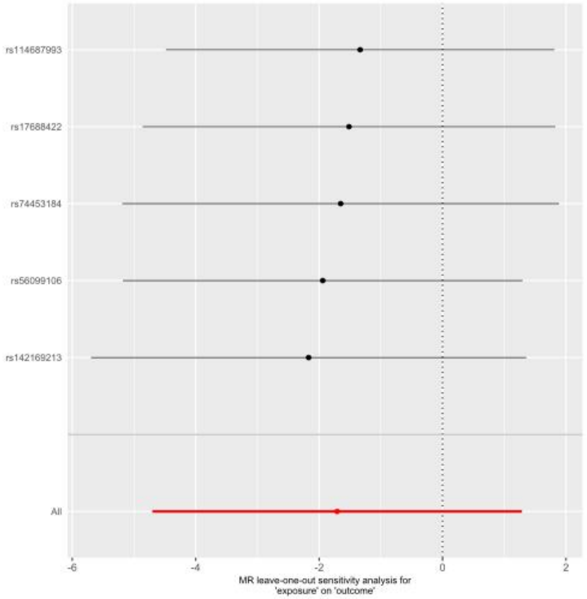

Pizza intake

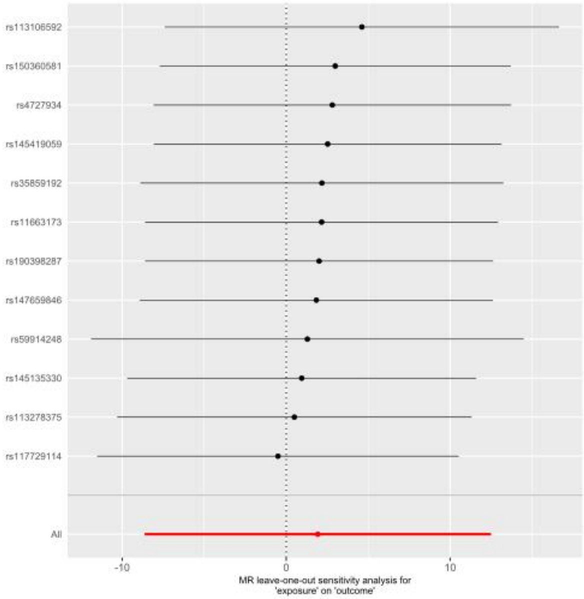

Snackpot intake

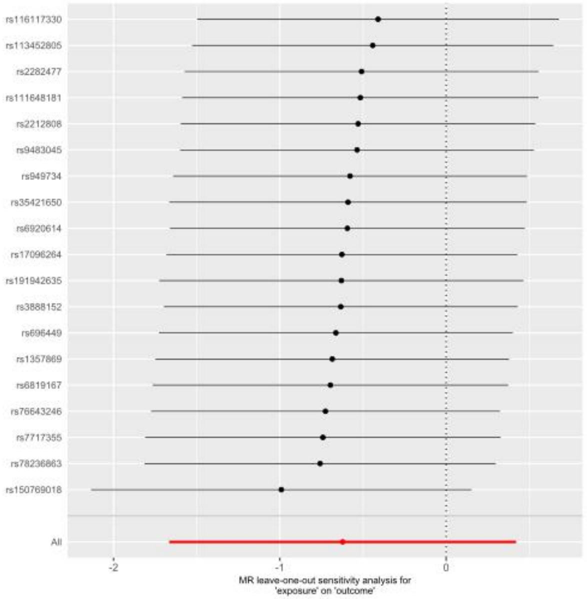

Fizzy drink intake

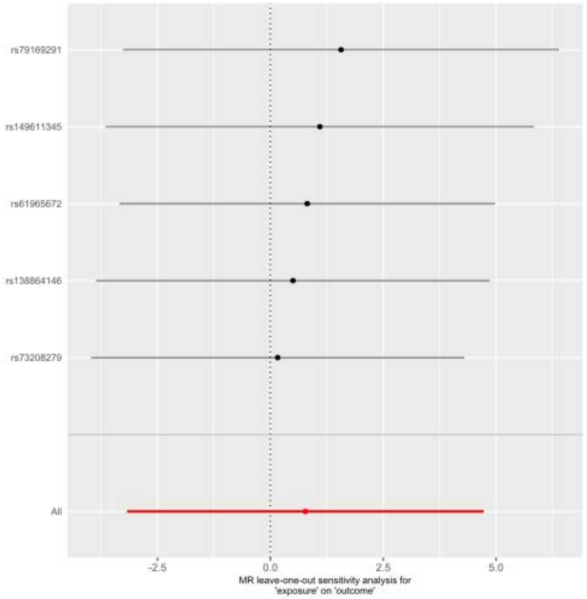

Flavoured milk intake

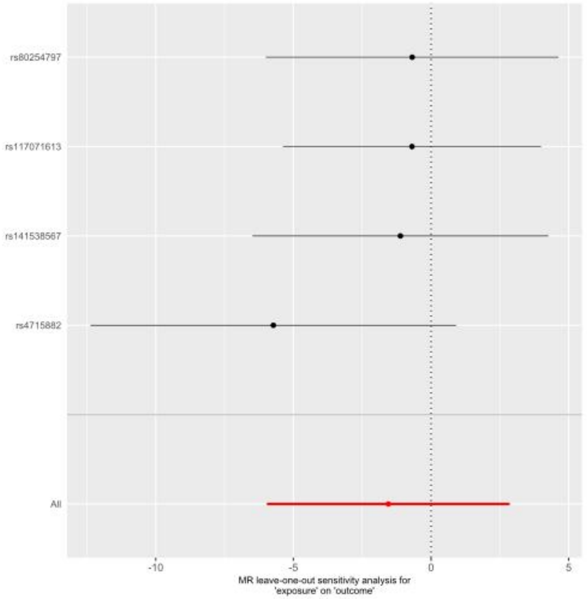

Grapefruit juice intake

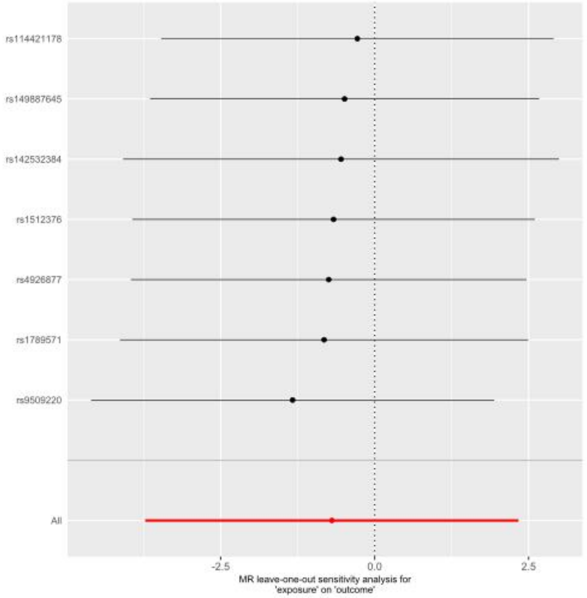

Hot chocolate intake

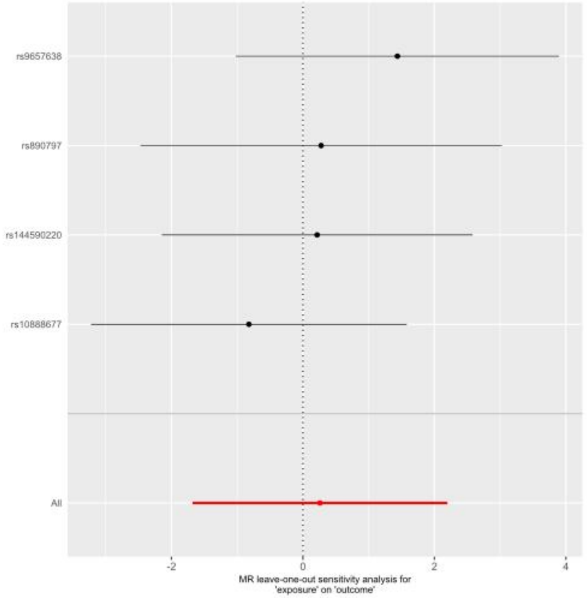

Low calorie drink intake

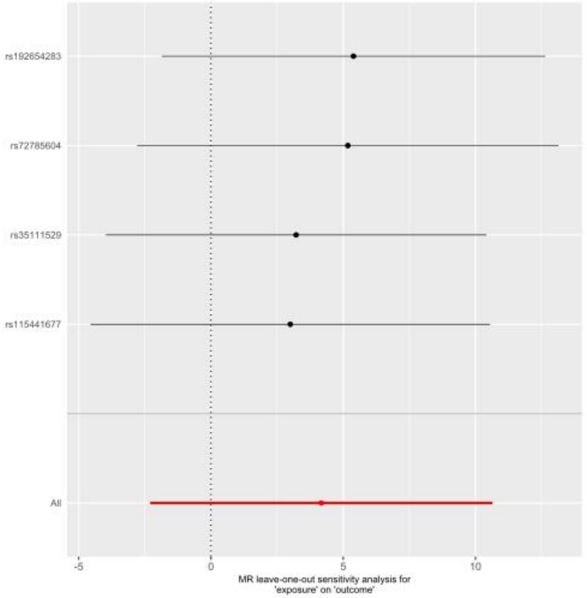

Low calorie hot chocolate intake

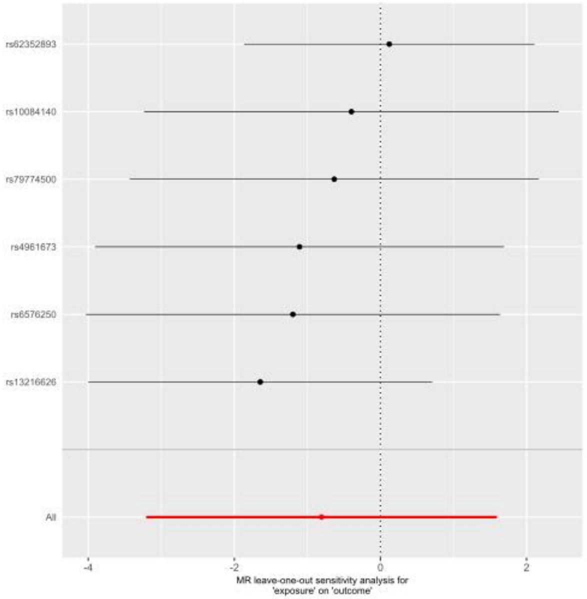

Orange juice intake

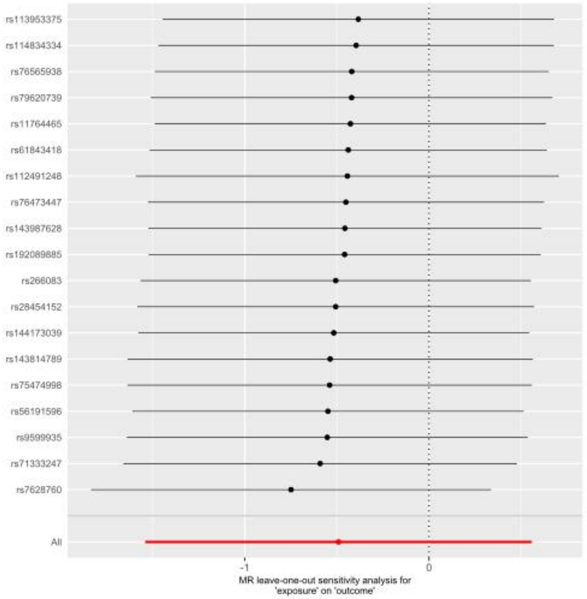

Pure fruitvegetable juice intake

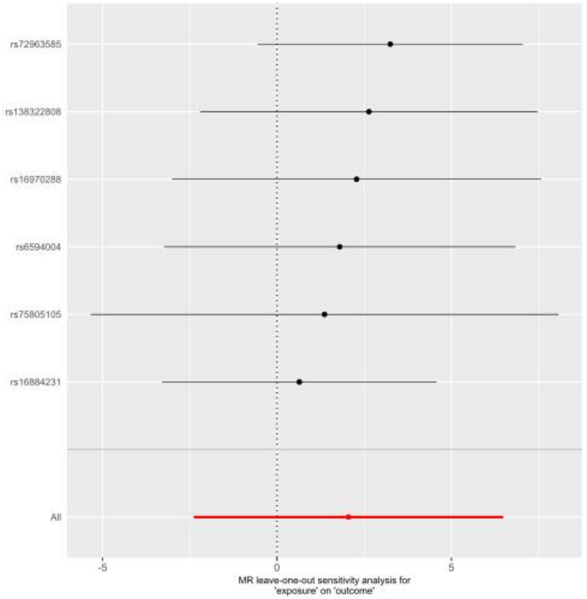

Other drink intake

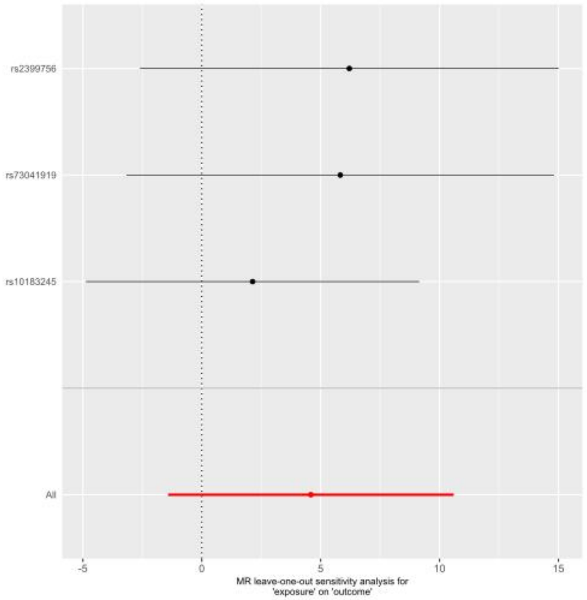

Other non-alcoholic drinks

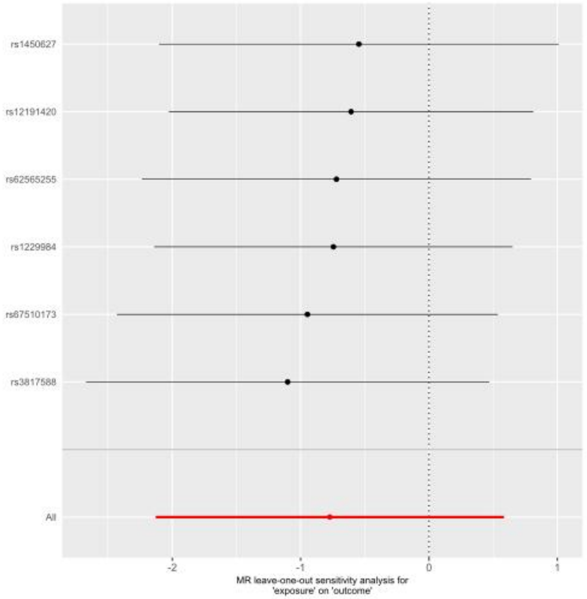

Beercider intake

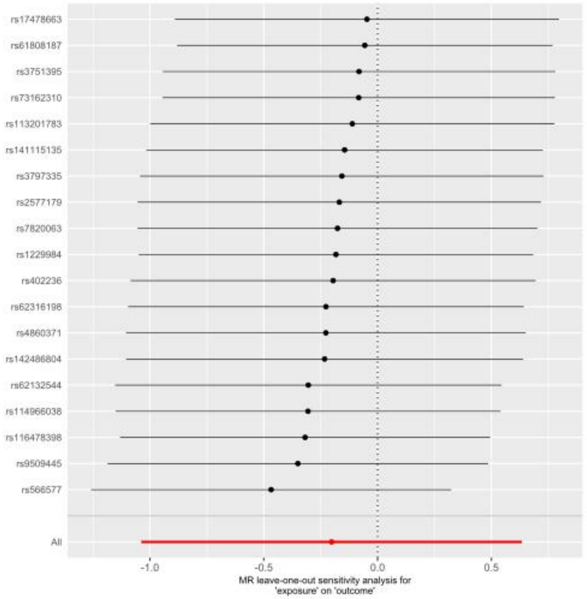

Red wine intake

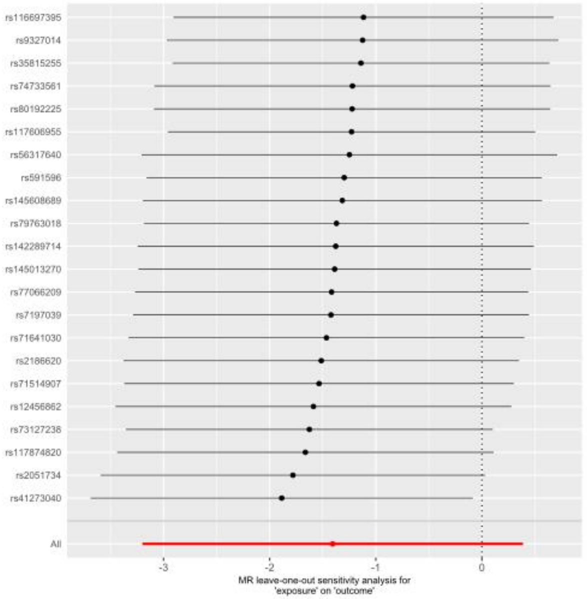

Rose wine intake

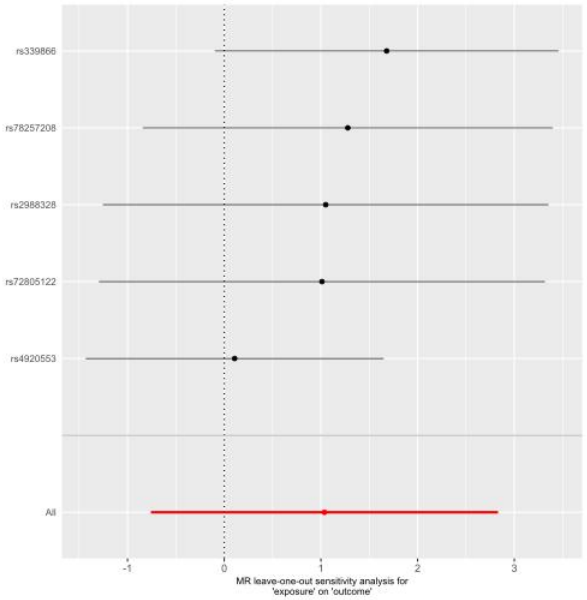

White wine intake

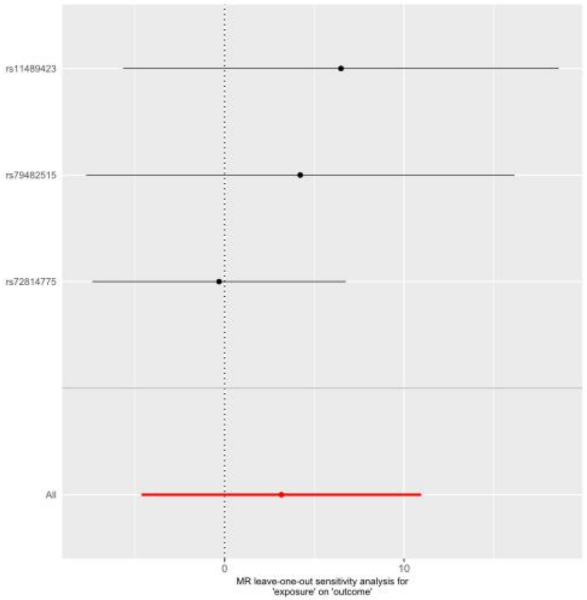

Fortified wine intake

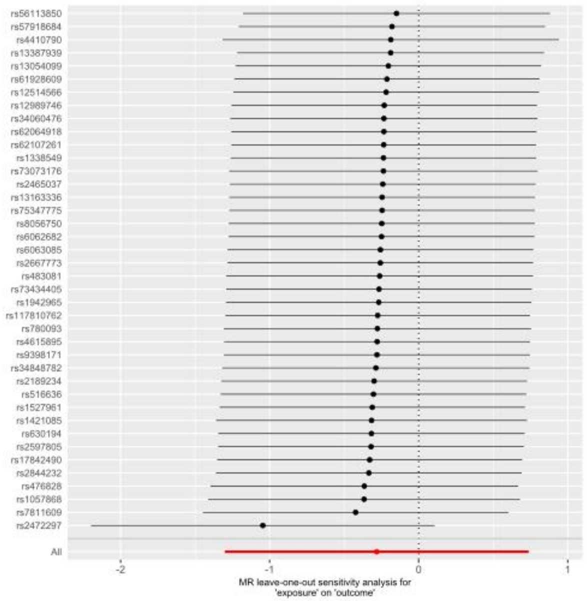

Coffee intake

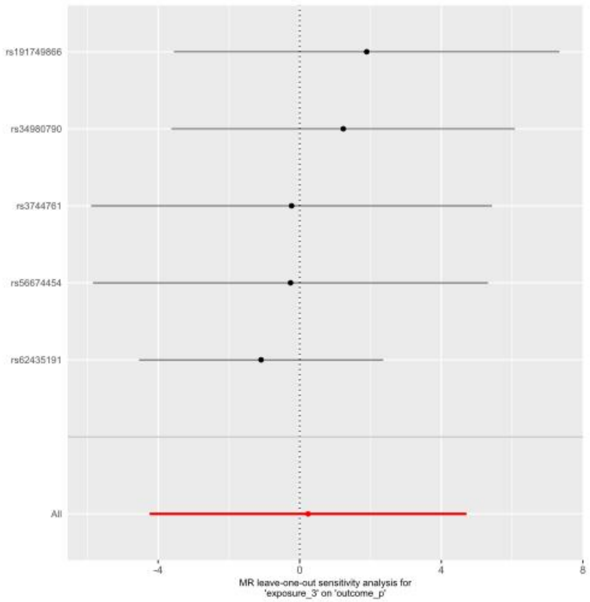

Milk intake

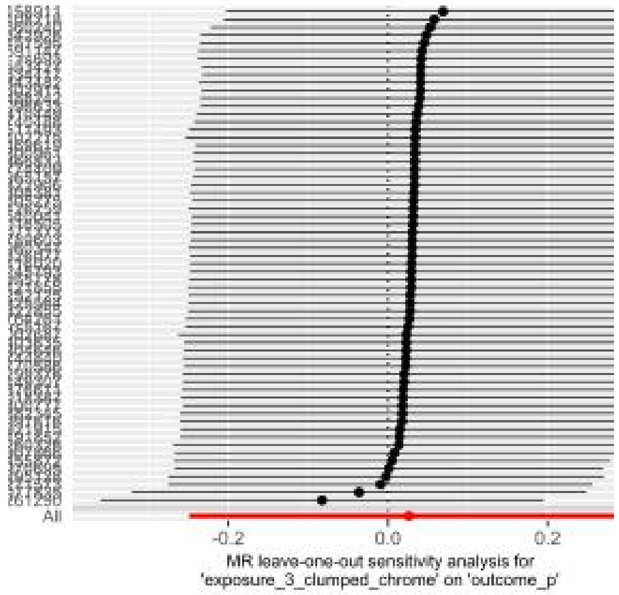

Polyunsaturated fatty acid levels

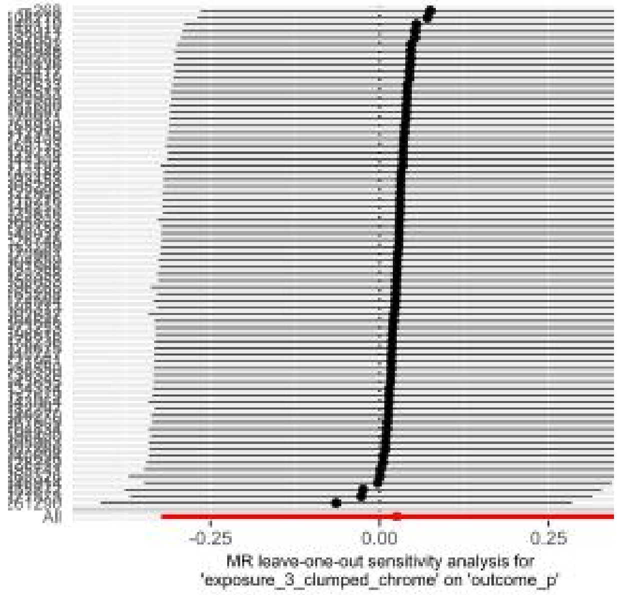

Monounsaturated fatty acid levels

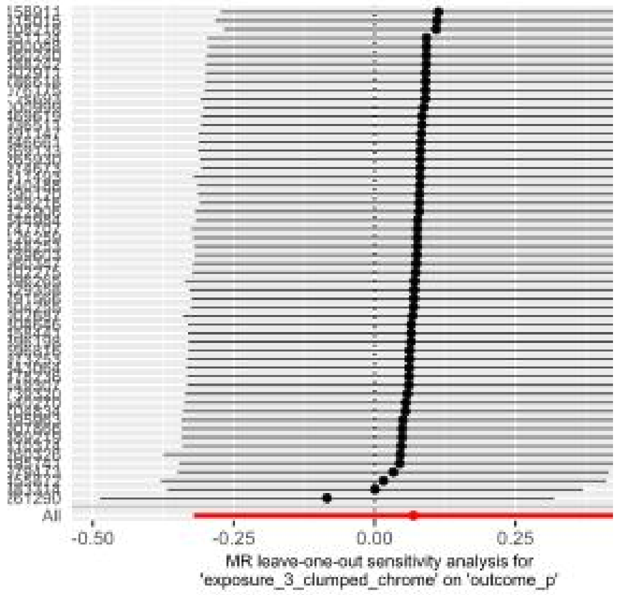

Saturated fatty acid levels

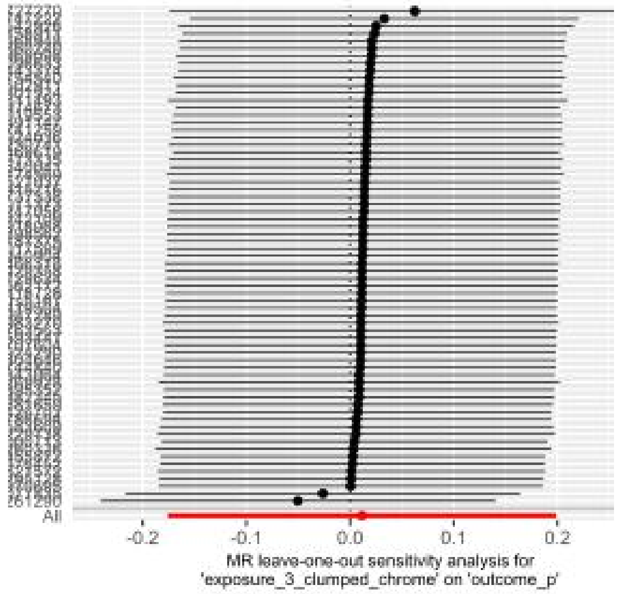

Omega-3 fatty acid levels

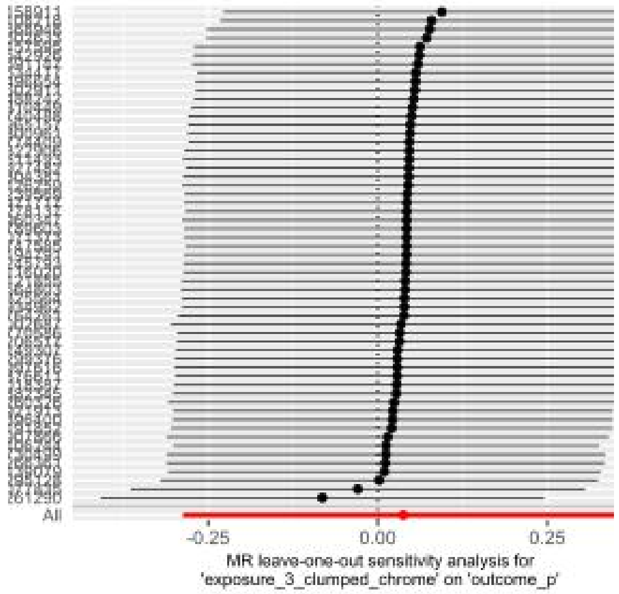

Omega-6 fatty acid levels

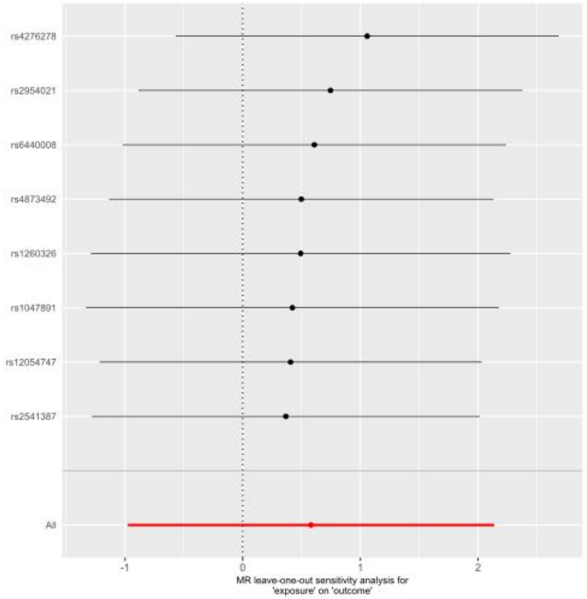

UNa/UCr

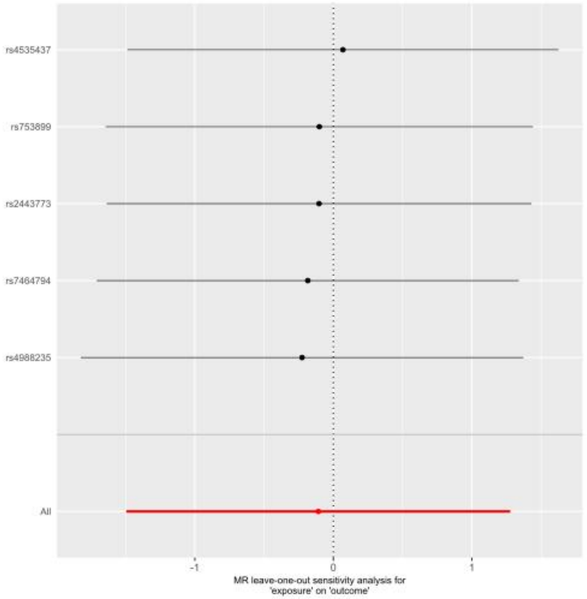

Ca

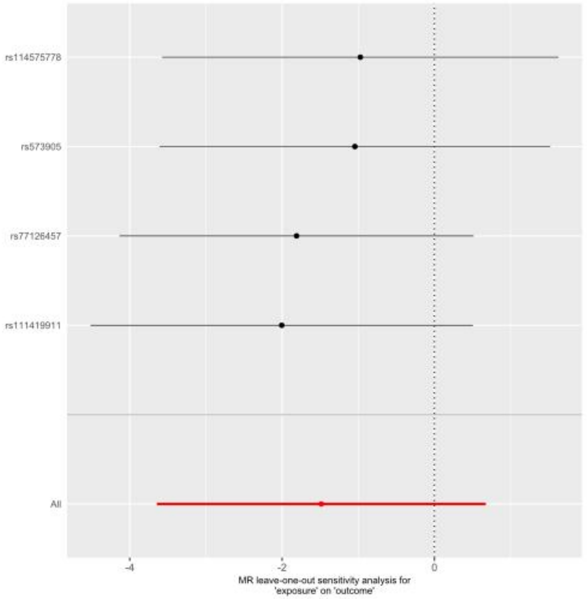

Mg

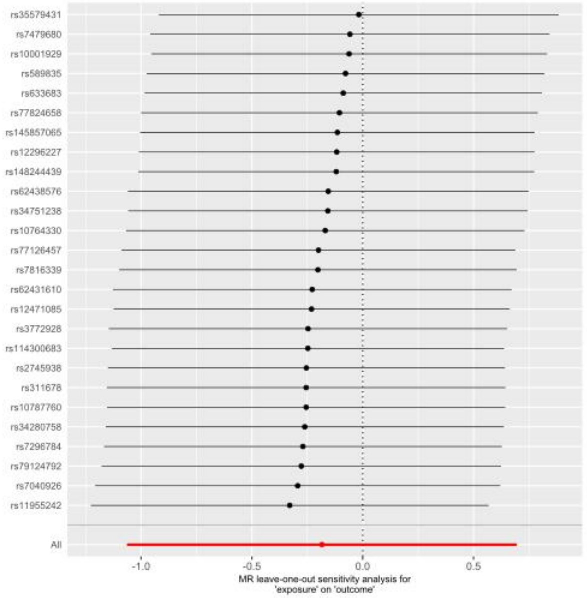

K

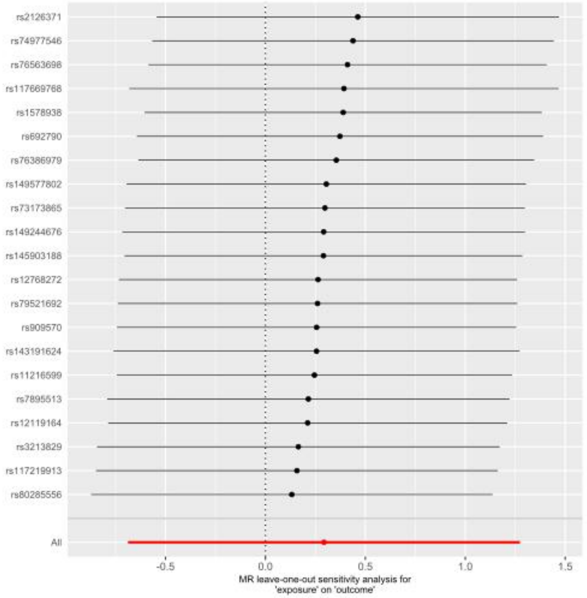

VA

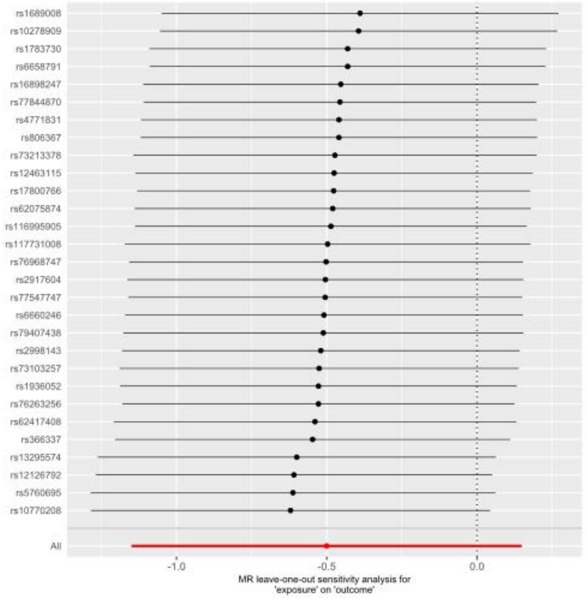

Carotene

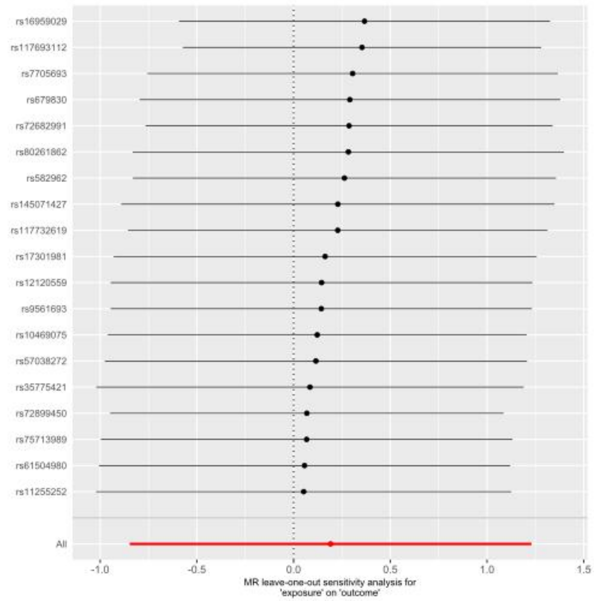

VD

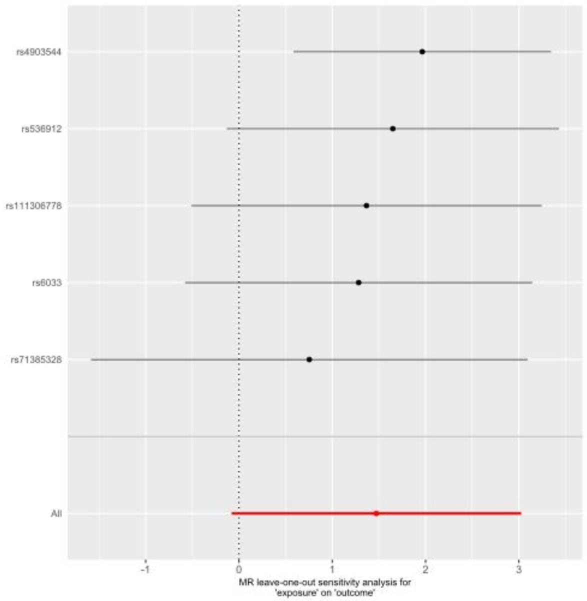

VE

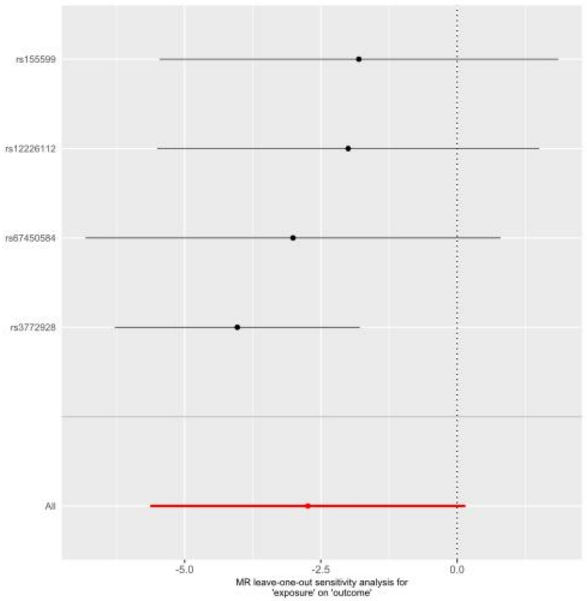

VB6

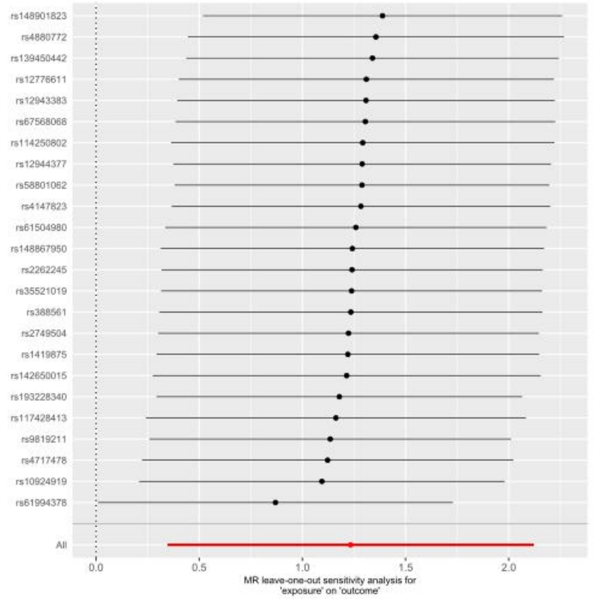

VB12

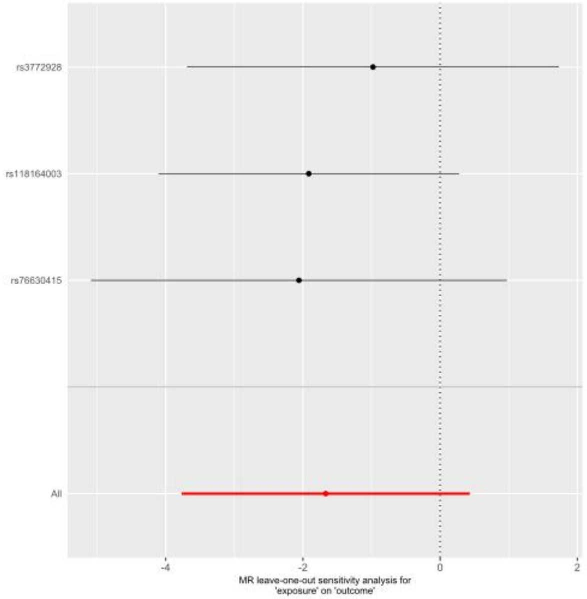

Folate

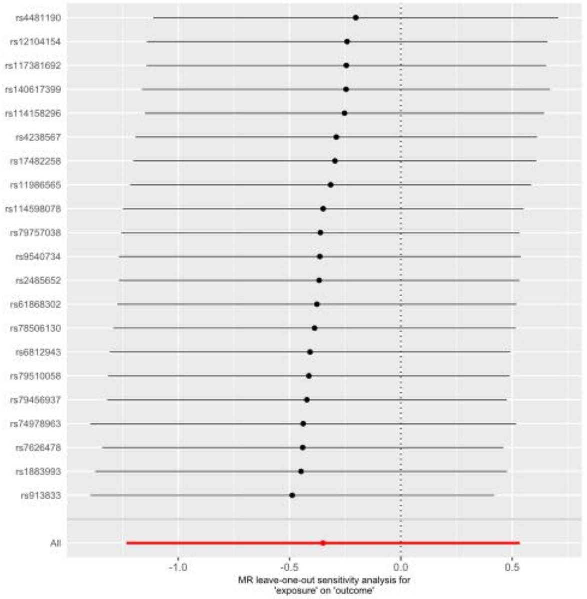

VC
