# Supplementary material for: The Combined Effects of the Most Important Dietary Patterns on the Incidence and Prevalence of Chronic Renal Failure: Results from the US National Health and Nutrition Examination Survey and Mendelian Analyses
Source: Nutrients. 2024 Jul 12;16(14):2248. doi: 10.3390/nu16142248 (PMC11280344; doi:10.3390/nu16142248)
Supplement: Supplementary file 1 [file nutrients-16-02248-s001.zip › Table S1.pdf]

Table S1. Food Composition of Dietary Pattern Scores.

Dietary Pattern	Food Composition
HEI-2020	whole fruits, green vegetables, beans, whole grains, dairy foods, total protein foods, seafood, plant proteins, fatty acids, refined grains, sodium, added sugars, and saturated fats
DII	carbohydrates, protein, total fat, alcohol, fiber, cholesterol, saturated fatty acids, monounsaturated fatty acids, polyunsaturated fatty acids, n-3 fatty acids, n-6 fatty acids, niacin, vitamin(V) A, thiamine, VB2, VB6, VB12, VC, VD, VE, iron, magnesium, zinc, selenium, folate, carotene, caffeine, and energy
aMed	vegetables, legumes, fruits, nuts, whole grains, red and processed meats, fish, alcohol, and a monounsaturated/saturated fat ratio
DASH	protein, fiber, magnesium, calcium, potassium, total fat, saturated fat, cholesterol, and sodium
