# Supplementary material for: The Combined Effects of the Most Important Dietary Patterns on the Incidence and Prevalence of Chronic Renal Failure: Results from the US National Health and Nutrition Examination Survey and Mendelian Analyses
Source: Nutrients. 2024 Jul 12;16(14):2248. doi: 10.3390/nu16142248 (PMC11280344; doi:10.3390/nu16142248)
Supplement: Supplementary file 1 [file nutrients-16-02248-s001.zip › Table S2.pdf]

Table S2. Characteristics of the GWAS summary data.

Exposure	Ethnicity	Consortium	Total Population	Unit	PMID	Identified SNPs[a]	*P*(GWAS)
Vegetable and fruit							
Dried fruit intake	European	MRC-IEU	421,764	SD	NA	37	5.00E-08
Fresh fruit intake	European	MRC-IEU	446,462	SD	NA	50	5.00E-08
Cooked vegetable intake	European	MRC-IEU	448,651	SD	NA	15	5.00E-08
Salad/raw vegetable intake	European	MRC-IEU	435,435	SD	NA	12	5.00E-08
Grain							
Cereal intake	European	MRC-IEU	441,640	SD	NA	35	5.00E-08
White rice intake	European	MRC-IEU	64,949	SD	NA	3	1.00E-06
Bread intake	European	MRC-IEU	452,236	SD	NA	29	5.00E-08
Whole-wheat cereal intake	European	MRC-IEU	64,949	SD	NA	18	1.00E-05
Englyst dietary fibre	European	MRC-IEU	64,979	SD	NA	7	1.00E-06
Nut and legume							
Salted nuts intake	European	MRC-IEU	64,949	SD	NA	6	1.00E-06
Unsalted nuts intake	European	MRC-IEU	64,949	SD	NA	23	1.00E-05
Salted peanuts intake	European	MRC-IEU	64,949	SD	NA	3	1.00E-06
Unsalted peanuts intake	European	MRC-IEU	64,949	SD	NA	4	5.00E-08
Broad bean intake	European	MRC-IEU	64,949	SD	NA	3	1.00E-06
Baked bean intake	European	MRC-IEU	64,949	SD	NA	3	1.00E-06
Green bean intake	European	MRC-IEU	64,949	SD	NA	3	1.00E-06
Total meat							
Beef intake	European	MRC-IEU	461,053	SD	NA	12	5.00E-08
Pork intake	European	MRC-IEU	460,162	SD	NA	12	5.00E-08
Lamb intake	European	MRC-IEU	64,942	SD	NA	22	1.00E-05
Processed meat intake	European	MRC-IEU	461,981	SD	NA	21	5.00E-08

Poultry intake	European	MRC-IEU	461,900	SD	NA	8	5.00E-08
Whole egg intake	European	MRC-IEU	64,949	SD	NA	7	1.00E-05
Non-oily fish intake	European	MRC-IEU	460,880	SD	NA	11	5.00E-08
Oily fish intake	European	MRC-IEU	460,443	SD	NA	55	5.00E-08
Ultra-processed food							
Sweets intake	European	MRC-IEU	64,949	SD	NA	5	1.00E-06
Soya dessert intake	European	MRC-IEU	64,947	SD	NA	8	1.00E-06
Fried potatoes intake	European	MRC-IEU	64,949	SD	NA	4	1.00E-06
Indian snacks intake	European	MRC-IEU	64,949	SD	NA	5	5.00E-08
Other dessert intake	European	MRC-IEU	64,949	SD	NA	5	1.00E-06
Pizza intake	European	MRC-IEU	64,949	SD	NA	5	1.00E-06
Snackpot intake	European	MRC-IEU	64,949	SD	NA	12	5.00E-08
Beverage and juice							
Fizzy drink intake	European	MRC-IEU	64,949	SD	NA	18	1.00E-05
Flavoured milk intake	European	MRC-IEU	64,941	SD	NA	4	1.00E-06
Grapefruit juice intake	European	MRC-IEU	64,949	SD	NA	4	1.00E-06
Hot chocolate intake	European	MRC-IEU	64,936	SD	NA	7	1.00E-06
Low calorie drink intake	European	MRC-IEU	64,949	SD	NA	4	1.00E-06
Low calorie hot chocolate intake	European	MRC-IEU	64,942	SD	NA	4	1.00E-06
Orange juice intake	European	MRC-IEU	64,949	SD	NA	6	1.00E-06
Pure fruitvegetable juice intake	European	MRC-IEU	64,949	SD	NA	20	1.00E-05
Other drink intake	European	MRC-IEU	64,949	SD	NA	6	1.00E-06
Other non-alcoholic drinks	European	MRC-IEU	64,949	SD	NA	3	1.00E-06
Alcohol							
Beercider intake	European	MRC-IEU	64,949	SD	NA	7	1.00E-06
Red wine intake	European	MRC-IEU	64,949	SD	NA	19	1.00E-05

Rose wine intake	European	MRC-IEU	64,949	SD	NA	22	1.00E-05
White wine intake	European	MRC-IEU	64,949	SD	NA	6	1.00E-06
Fortified wine intake	European	MRC-IEU	64,942	SD	NA	4	1.00E-07
Water and tea and coffee							
Coffee intake	European	MRC-IEU	428,860	SD	NA	40	5.00E-08
Dairy product							
Milk intake	European	MRC-IEU	64,943	SD	NA	5	1.00E-06
Fatty acid							
Polyunsaturated fatty acid levels	European	NA	115,006	SD	35213538	65	5.00E-08
Monounsaturated fatty acid levels	European	NA	115,006	SD	35213538	75	5.00E-08
Saturated fatty acid levels	European	NA	115,006	SD	35213538	58	5.00E-08
Omega-3 fatty acid levels	European	NA	115,006	SD	35213538	69	5.00E-08
Omega-6 fatty acid levels	European	NA	115,006	SD	35213538	61	5.00E-08
Sodium intake							
UNa/UCr	European	NA	327,616	SD	30910378	9	5.00E-08
Microelement							
Ca	European	MRC-IEU	64,979	SD	NA	5	1.00E-06
Mg	European	MRC-IEU	64,979	SD	NA	4	1.00E-06
K	European	MRC-IEU	64,979	SD	NA	27	1.00E-05
VA	European	MRC-IEU	62,991	SD	NA	20	1.00E-05
Carotene	European	MRC-IEU	64,979	SD	NA	28	1.00E-05
VD	European	MRC-IEU	64,979	SD	NA	23	1.00E-05
VE	European	MRC-IEU	64,979	SD	NA	5	1.00E-06
VB6	European	MRC-IEU	64,979	SD	NA	4	1.00E-06
VB12	European	MRC-IEU	64,979	SD	NA	23	1.00E-05
Folate	European	MRC-IEU	64,979	SD	NA	3	1.00E-06

VC	European	MRC-IEU	64,979	SD	NA	22	1.00E-05

Abbreviations: NA, not available; GWAS, genome-wide association study; logOR, logarithm of odds ratio; MRC-IEU, Medical Research Council Integrative Epidemiology Unit; PMID, Pubmed Unique Identifier; SNP, single nucleotide polymorphisms.
