# Supplementary material for: The Combined Effects of the Most Important Dietary Patterns on the Incidence and Prevalence of Chronic Renal Failure: Results from the US National Health and Nutrition Examination Survey and Mendelian Analyses
Source: Nutrients. 2024 Jul 12;16(14):2248. doi: 10.3390/nu16142248 (PMC11280344; doi:10.3390/nu16142248)
Supplement: Supplementary file 1 [file nutrients-16-02248-s001.zip › Table S3.pdf]

Table S3. Baseline characteristics of dietary patterns and nutrients: NHANES (2005 to 2020).

Characteristics	Total (N=25167)	Non-CKD (N=21006)	CKD (N=4161)	P-value
**Dietary patterns and nutrients, Mean(SE)**				
**Dietary patterns**				
HEI-2020	51.3(0.1)	51.3(0.1)	51.4(0.2)	0.515
DII	1.2(0.01)	1.1(0.01)	1.5(0.03)	<0.001
DASH	3.50(0.01)	3.51(0.01)	3.46(0.02)	0.013
aMed	5.7(0.01)	5.7(0.01)	5.7(0.01)	0.855
**Nutrients**				
Energy, kcal	2037.4(5.2)	2075.9(5.7)	1843.3(11.5)	<0.001
Protein, g	79.9(0.2)	81.3(0.2)	72.8(0.5)	<0.001
Carbohydrate, g	245.4(0.7)	250.3(0.7)	221.0(1.5)	<0.001
Total sugars, g	107.1(0.4)	109.4(0.4)	95.7(0.9)	<0.001
Total fat, g	78.5(0.2)	79.8(0.3)	71.9(0.5)	<0.001
Total saturated fatty acids, g	25.3(0.1)	25.7(0.1)	23.4(0.2)	<0.001
Total monounsaturated fatty acids, g	27.8(0.1)	28.3(0.1)	25.4(0.2)	<0.001
Total polyunsaturated fatty acids, g	18.1(0.1)	18.4(0.1)	16.5(0.1)	<0.001
Cholesterol, mg	292.4(1.2)	294.4(1.3)	282.4(2.9)	<0.001
Dietary fiber, g	16.8(0.1)	17.1(0.1)	15.3(0.1)	<0.001
Retinol, mcg	404.5(2.5)	403.3(2.6)	410.1(7.4)	0.321
Vitamin A, RAE, mcg	610.0(3.3)	610.8(3.6)	606.0(8.6)	0.585
Alpha-carotene, mcg	402.6(6.1)	405.5(6.9)	388.1(12.1)	0.207
Beta-carotene, mcg	2224.8(22.2)	2246.2(25.1)	2116.5(45.1)	0.012
Beta-cryptoxanthin, mcg	88.9(1.4)	89.0(1.5)	88.6(3.0)	0.904
Lycopene, mcg	4888.0(43.5)	5015.6(48.4)	4243.9(96.1)	<0.001
Thiamin (Vitamin B1), mg	1.6(0.0)	1.6(0.01)	1.5(0.01)	<0.001
Riboflavin (Vitamin B2), mg	2.0(0.01)	2.0(0.01)	1.9(0.01)	<0.001
Niacin (Vitamin B3), mg	24.9(0.1)	25.4(0.1)	22.3(0.2)	<0.001
Vitamin B6, mg	2.0(0.01)	2.1(0.01)	1.8(0.02)	<0.001
Total folate, mcg	388.5(1.3)	395.5(1.5)	352.9(2.9)	<0.001
Total choline, mg	326.3(1.0)	329.5(1.1)	310.2(2.4)	<0.001
Vitamin B12, mcg	4.9(0.03)	4.9(0.03)	4.7(0.1)	0.003
Vitamin C, mg	82.9(0.5)	83.8(0.5)	78.3(1.1)	<0.001
Vitamin D (D2 + D3), mcg	4.6(0.03)	4.6(0.03)	4.4(0.1)	0.017
Vitamin E as alpha-tocopherol, mg	8.2(0.03)	8.4(0.04)	7.5(0.1)	<0.001
Vitamin K, mcg	113.6(1.2)	115.4(1.3)	104.6(1.9)	<0.001
Calcium, mg	913.9(3.1)	929.7(3.4)	833.7(7.0)	<0.001
Phosphorus, mg	1325.0(3.6)	1347.9(4.0)	1209.2(8.0)	<0.001
Magnesium, mg	290.6(0.8)	296.0(0.9)	263.5(1.7)	<0.001
Iron, mg	14.4(0.1)	14.6(0.05)	13.5(0.1)	<0.001
Zinc, mg	10.9(0.04)	11.1(0.04)	10.1(0.1)	<0.001
Copper, mg	1.22(0.0)	1.2(0.01)	1.1(0.01)	<0.001
Sodium, mg	3374.8(9.3)	3433.0(10.3)	3081.2(20.9)	<0.001
Potassium, mg	2579.4(6.8)	2611.3(7.5)	2418.2(15.1)	0.004
Selenium, mcg	111.4(0.3)	113.2(0.4)	102.6(0.8)	<0.001

Note: Descriptive data were shown as mean (SE) while categorical variables were reported as n (%). P-values less than 0.05 (P-value < 0.05) were considered significant. Abbreviation: CKD,chronic kidney disease; HEI, Healthy eating index; DII, Dietary Inflammation Index; aMed, Alternate Mediterranean Diet; DASH, Dietary Approaches to Stop Hypertension; N, number.
