# Supplementary figures and images for: The Combined Effects of the Most Important Dietary Patterns on the Incidence and Prevalence of Chronic Renal Failure: Results from the US National Health and Nutrition Examination Survey and Mendelian Analyses
Source: Nutrients. 2024 Jul 12;16(14):2248. doi: 10.3390/nu16142248 (PMC11280344; doi:10.3390/nu16142248)

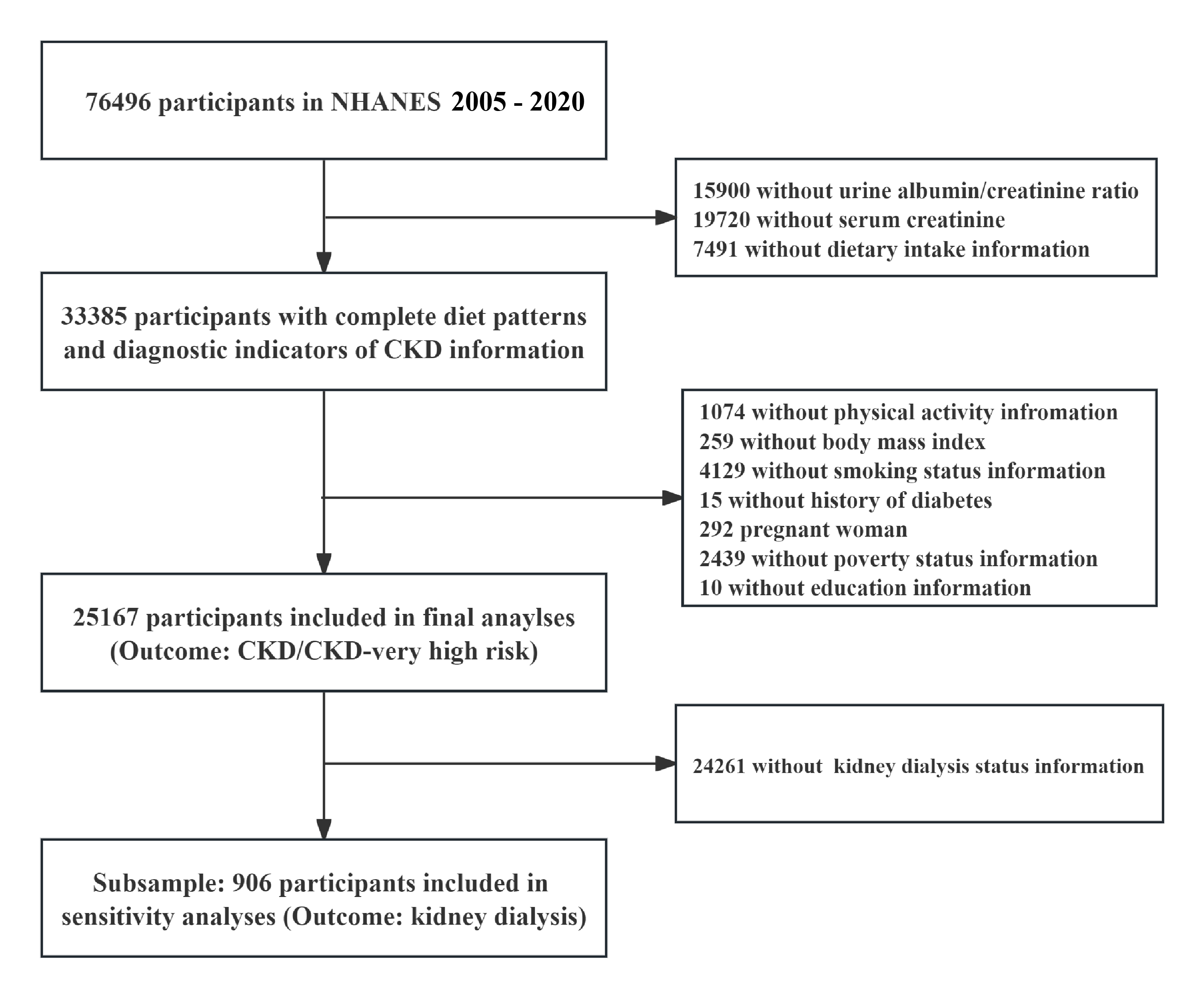

Supplement: Supplementary file 1 [file nutrients-16-02248-s001.zip › Figure S1.png]

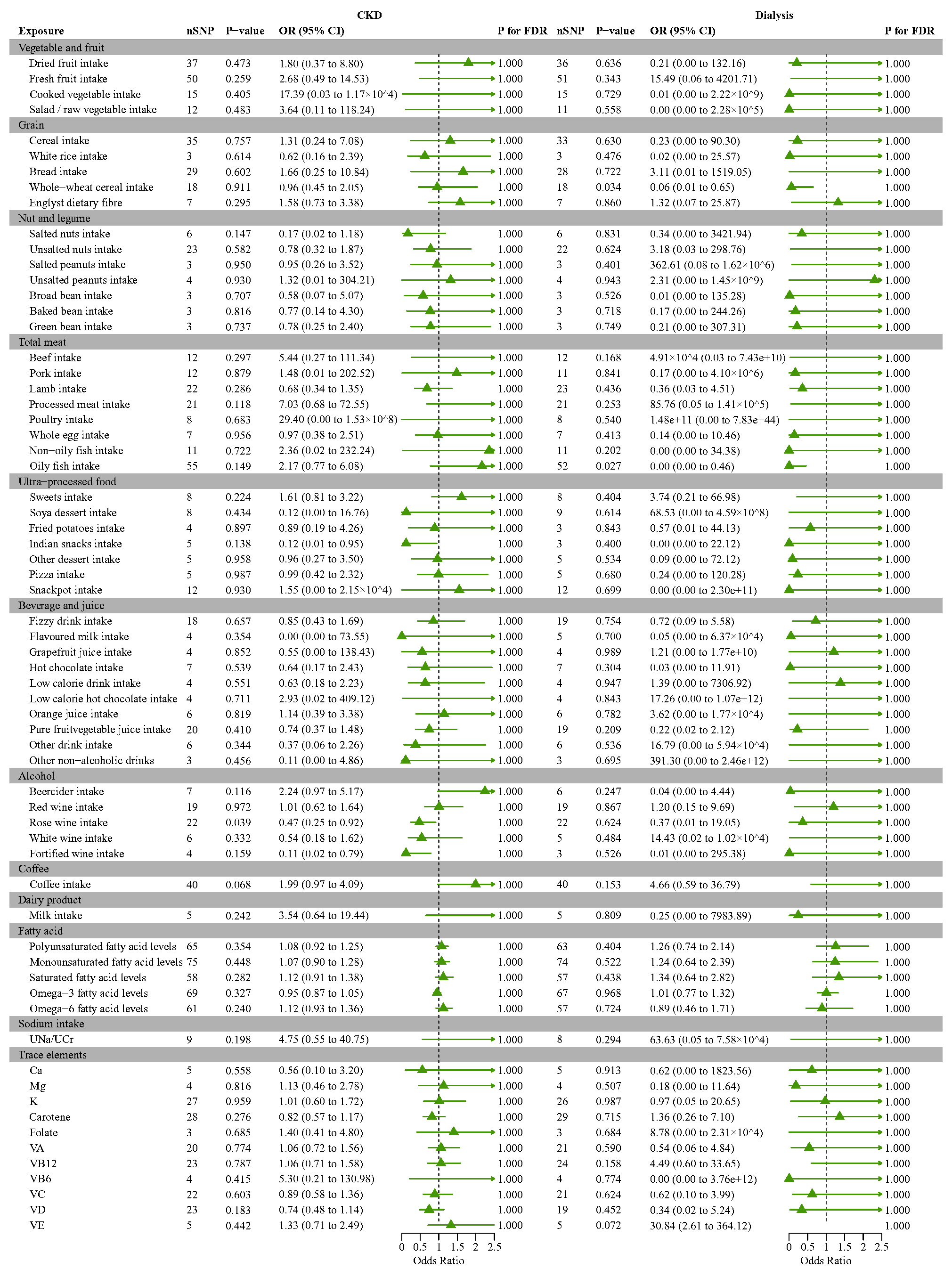

Supplement: Supplementary file 1 [file nutrients-16-02248-s001.zip › Figure S10.tif]

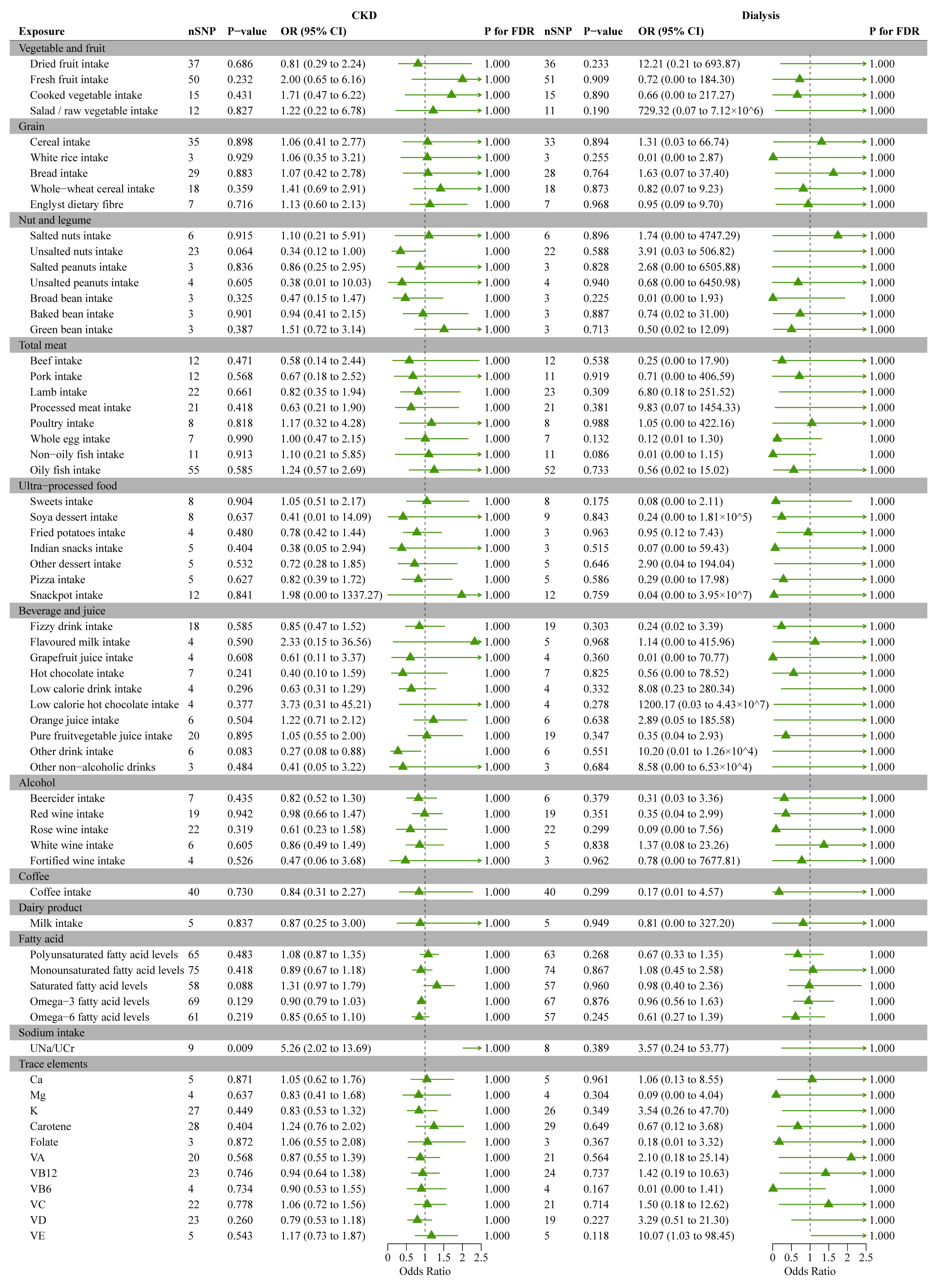

Supplement: Supplementary file 1 [file nutrients-16-02248-s001.zip › Figure S11.tif]

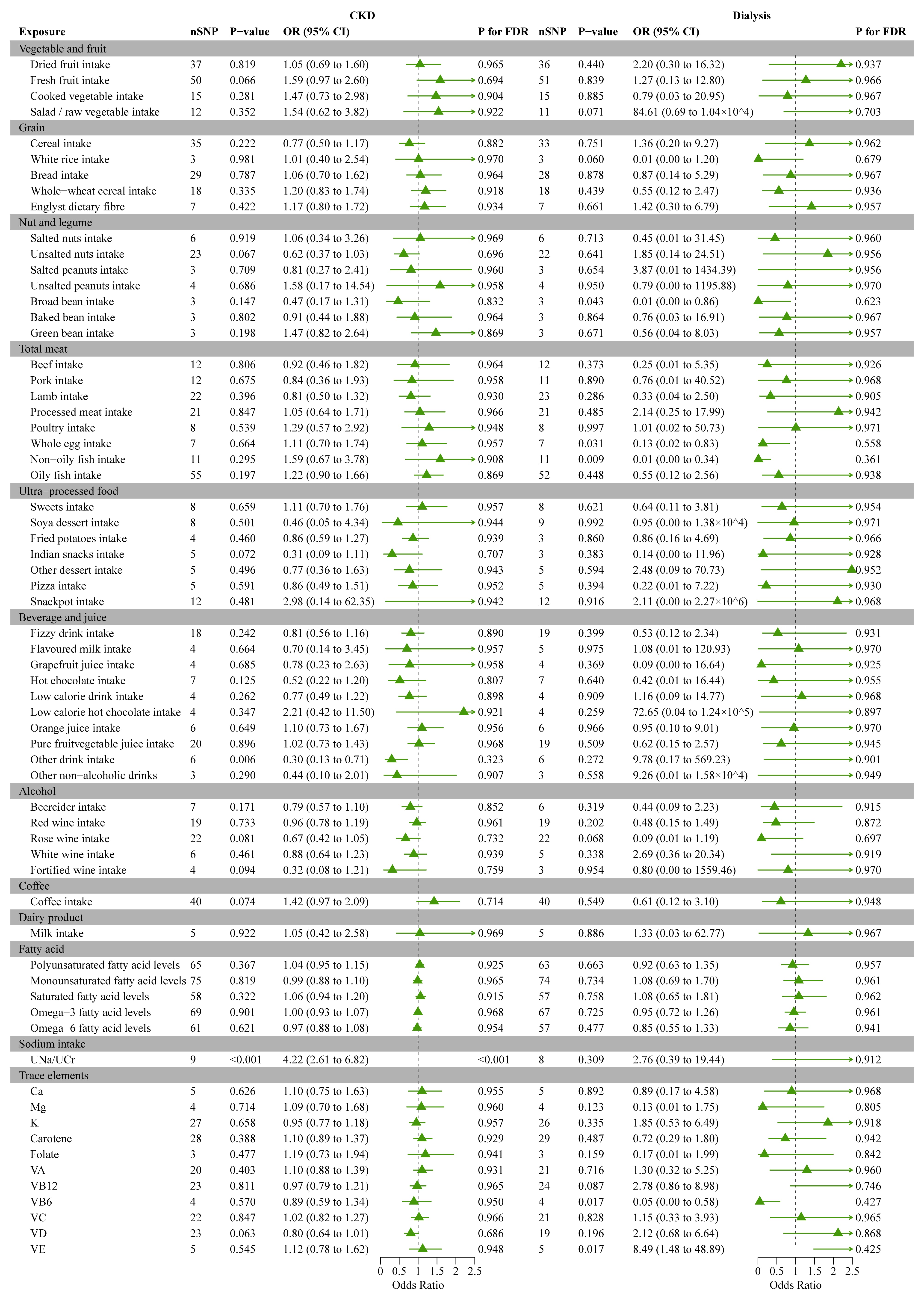

Supplement: Supplementary file 1 [file nutrients-16-02248-s001.zip › Figure S12.tif]

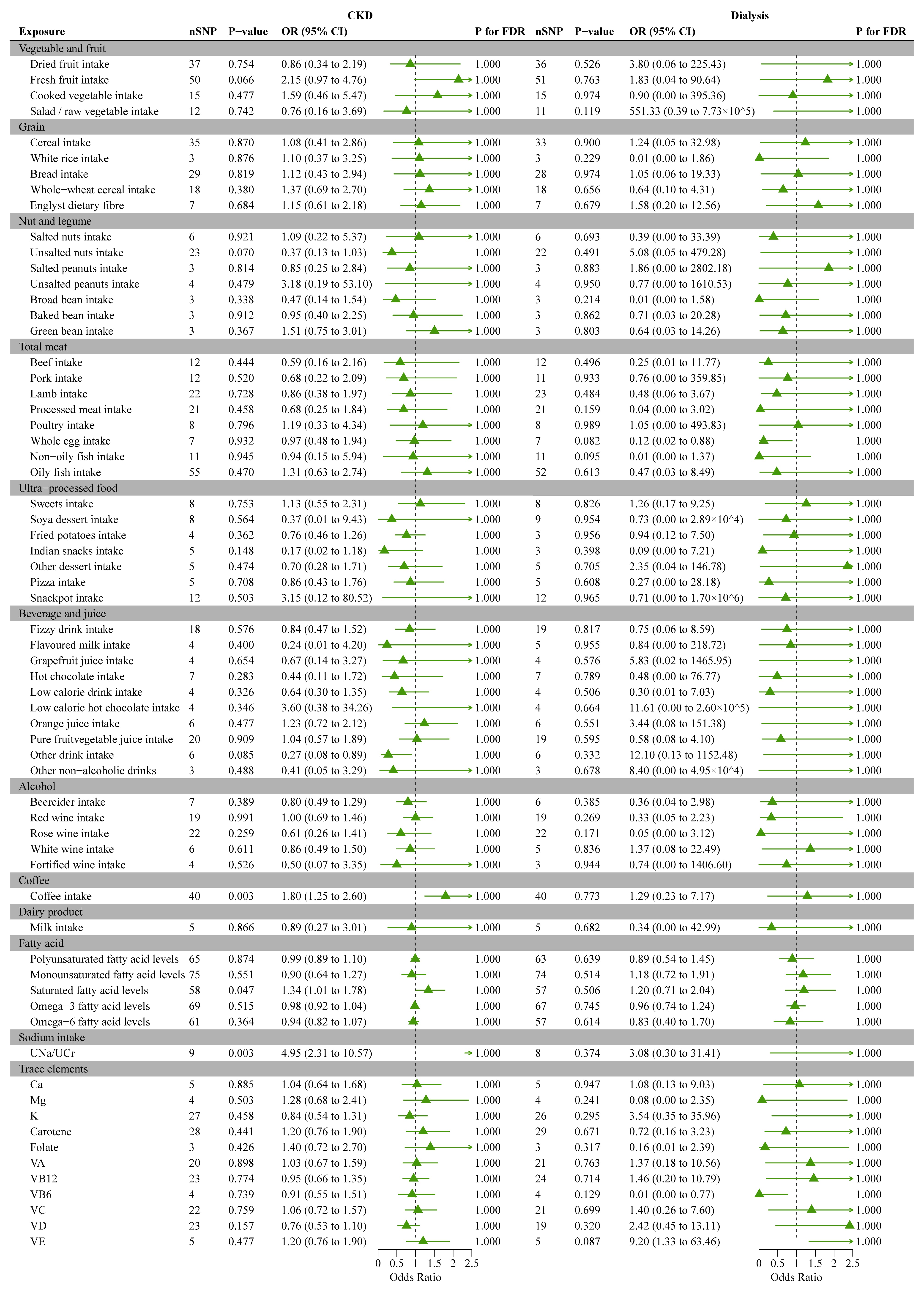

Supplement: Supplementary file 1 [file nutrients-16-02248-s001.zip › Figure S13.tif]

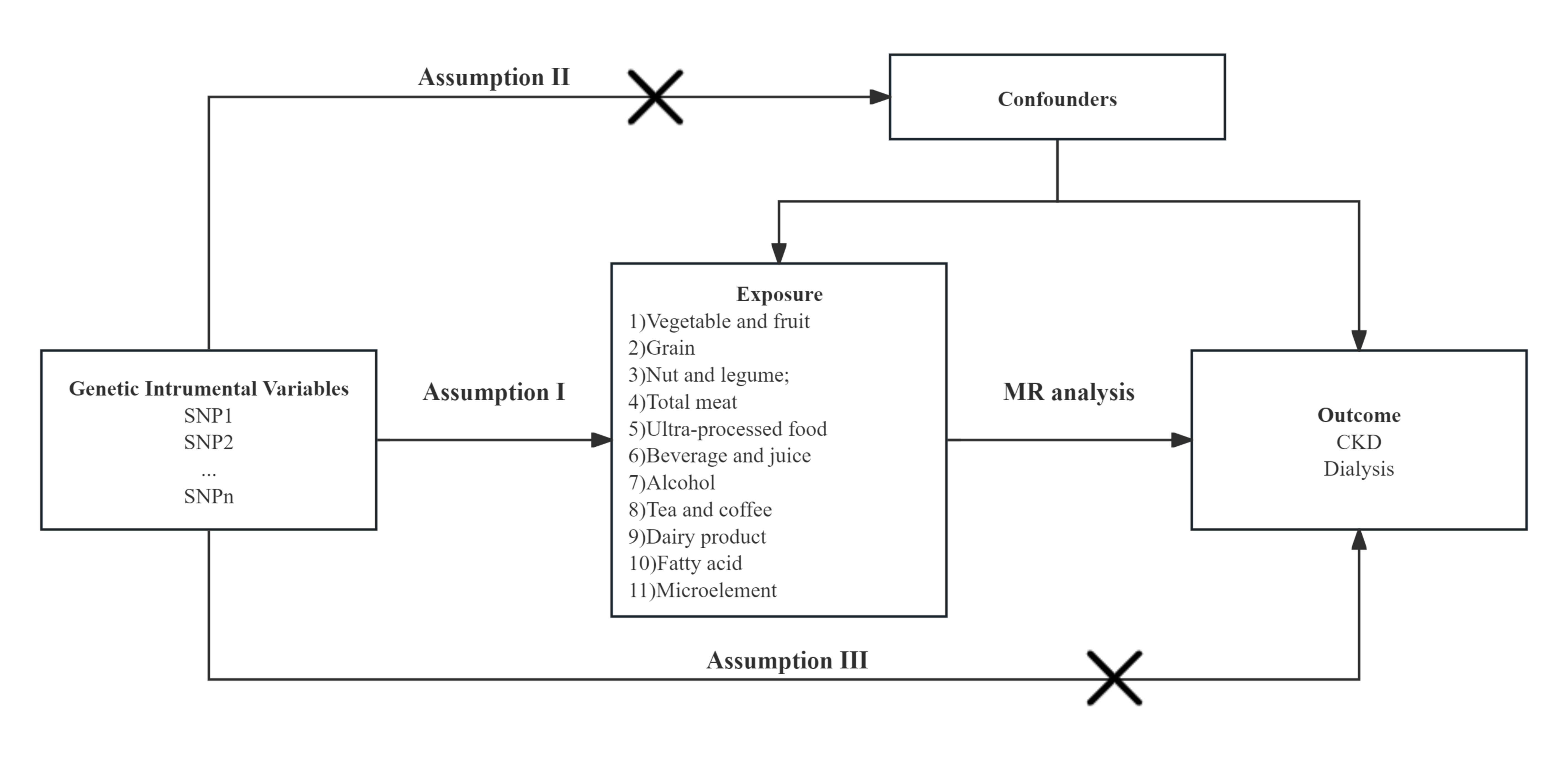

Supplement: Supplementary file 1 [file nutrients-16-02248-s001.zip › Figure S2.tif]

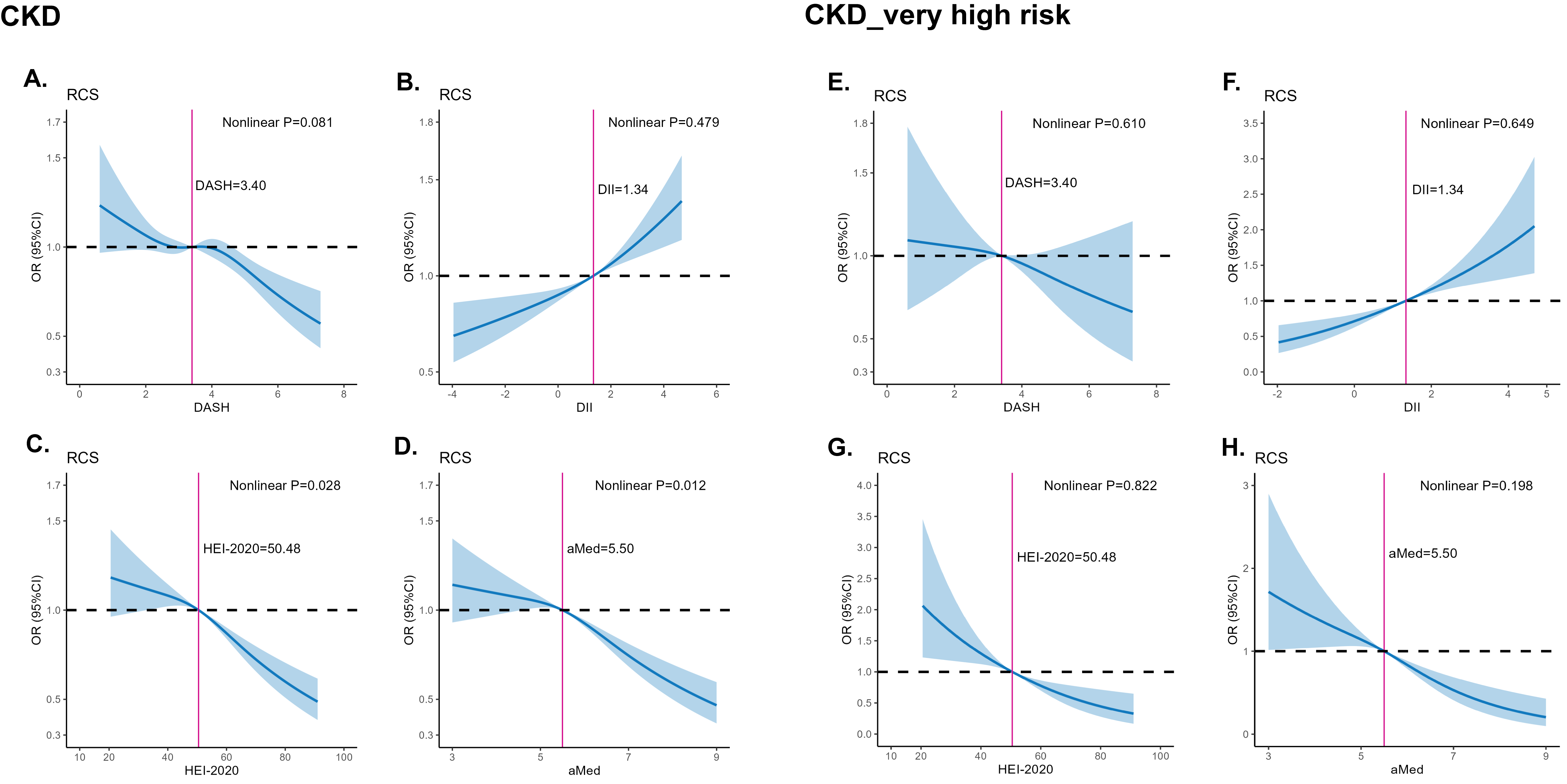

Supplement: Supplementary file 1 [file nutrients-16-02248-s001.zip › Figure S3.tif]

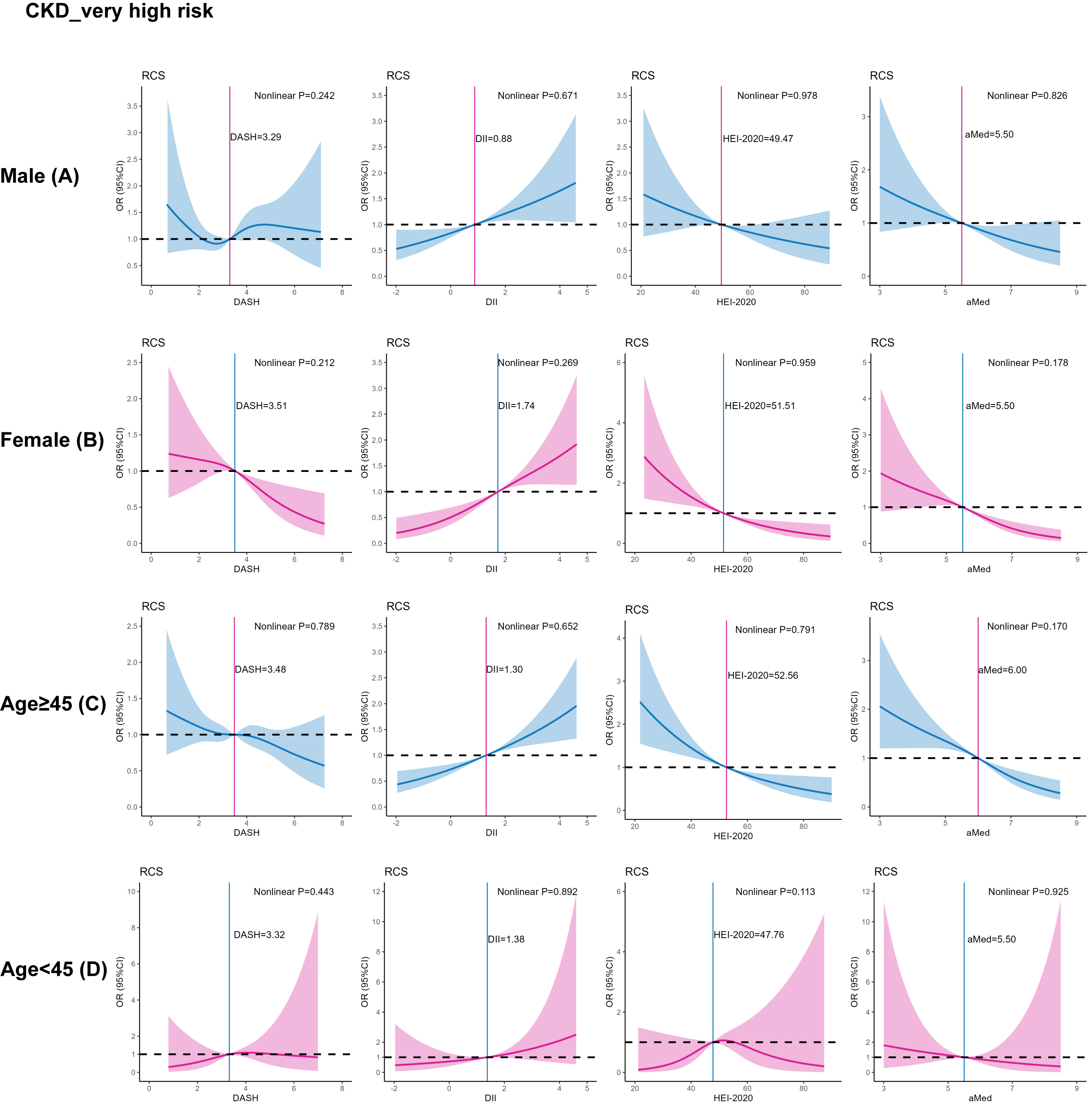

Supplement: Supplementary file 1 [file nutrients-16-02248-s001.zip › Figure S4.tif]

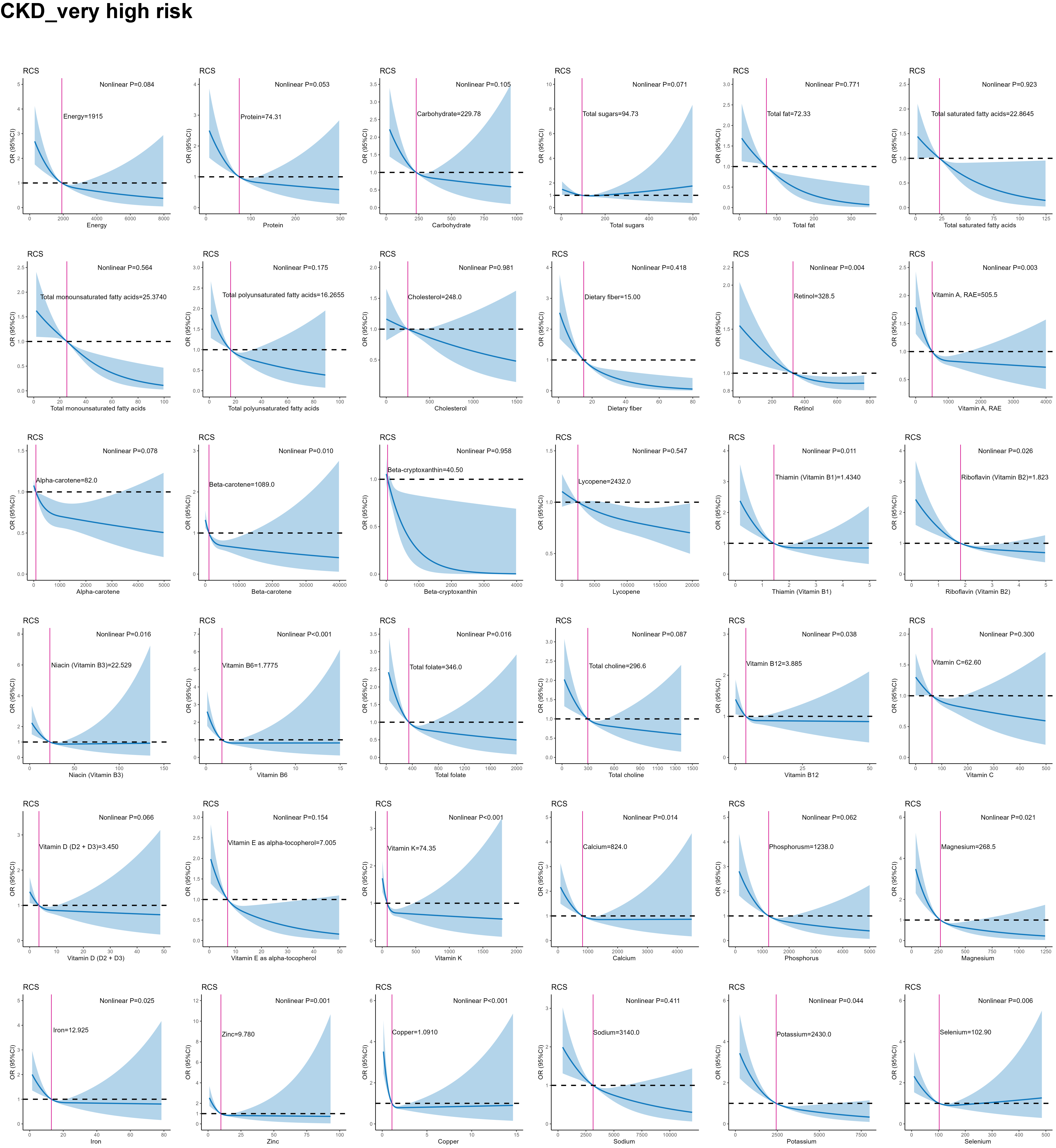

Supplement: Supplementary file 1 [file nutrients-16-02248-s001.zip › Figure S5.tif]

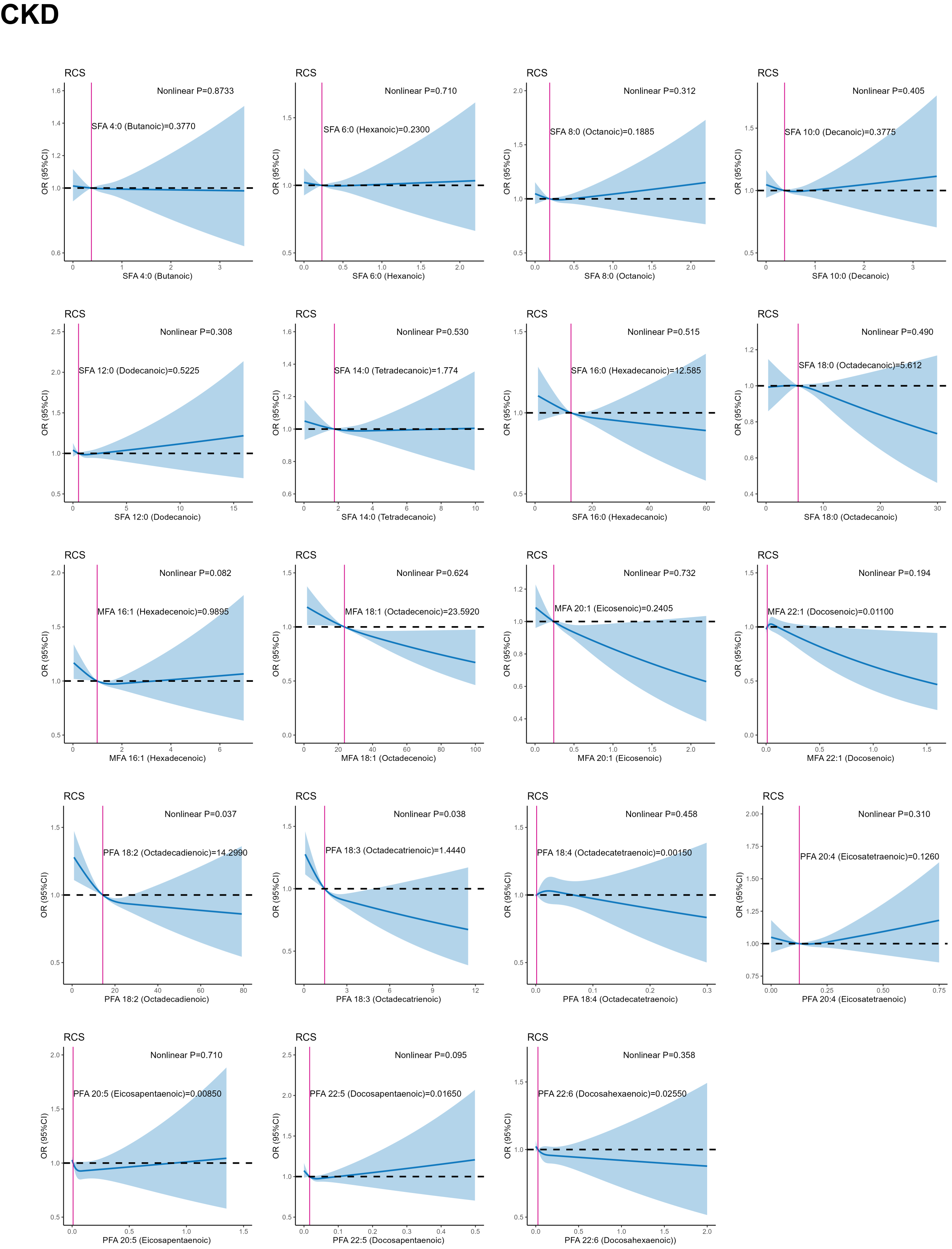

Supplement: Supplementary file 1 [file nutrients-16-02248-s001.zip › Figure S6.tif]

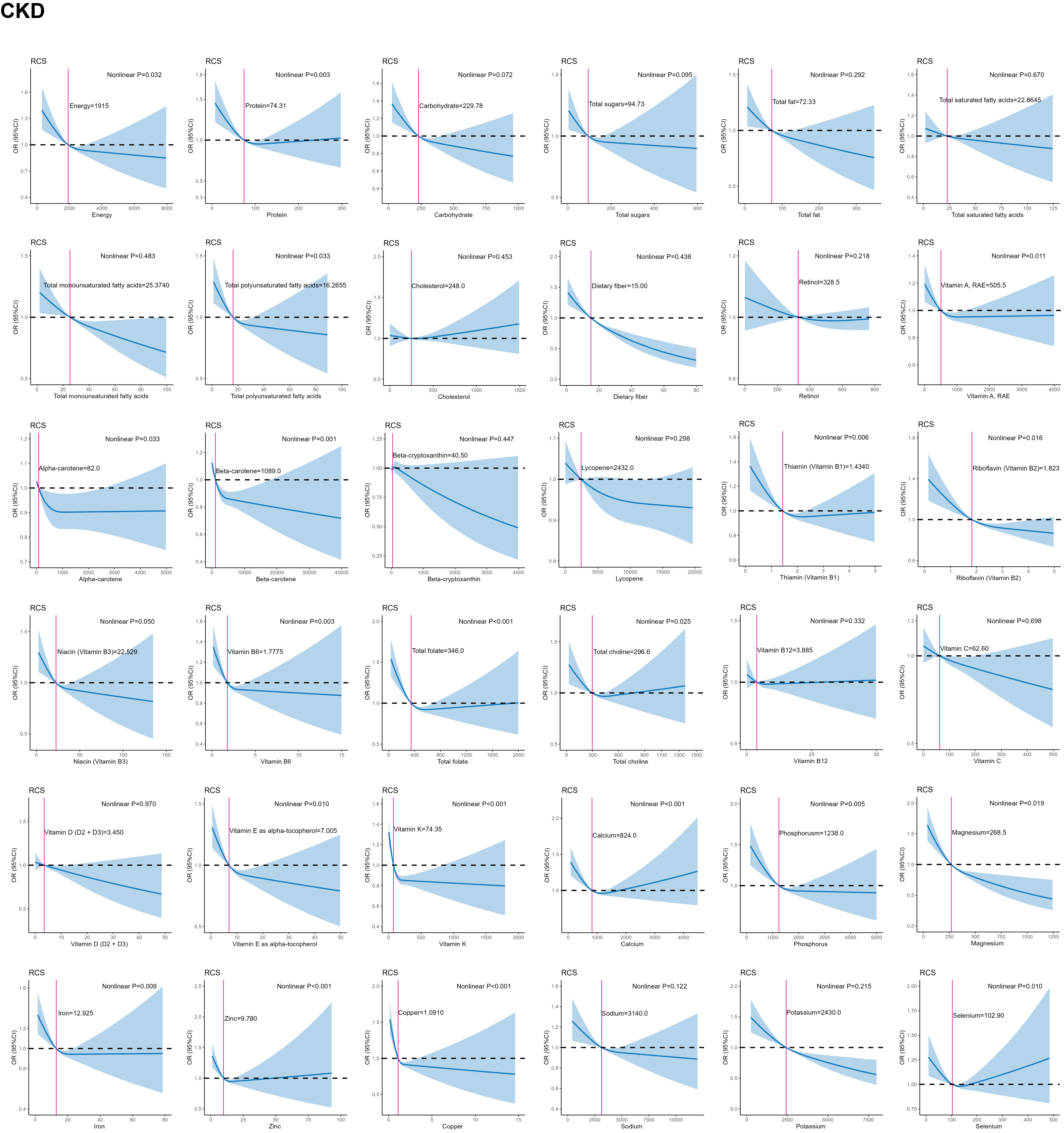

Supplement: Supplementary file 1 [file nutrients-16-02248-s001.zip › Figure S7.tif]

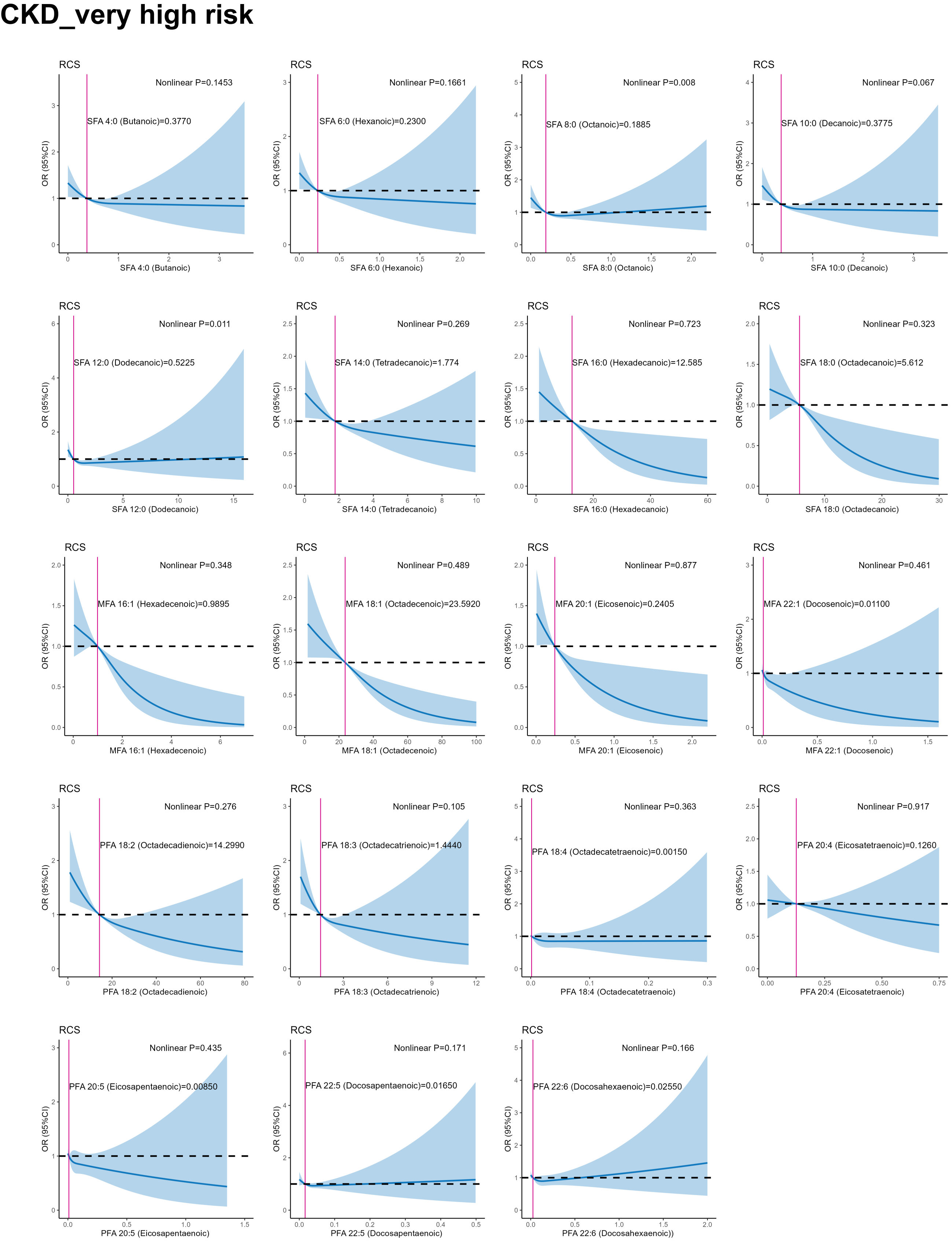

Supplement: Supplementary file 1 [file nutrients-16-02248-s001.zip › Figure S8.tif]

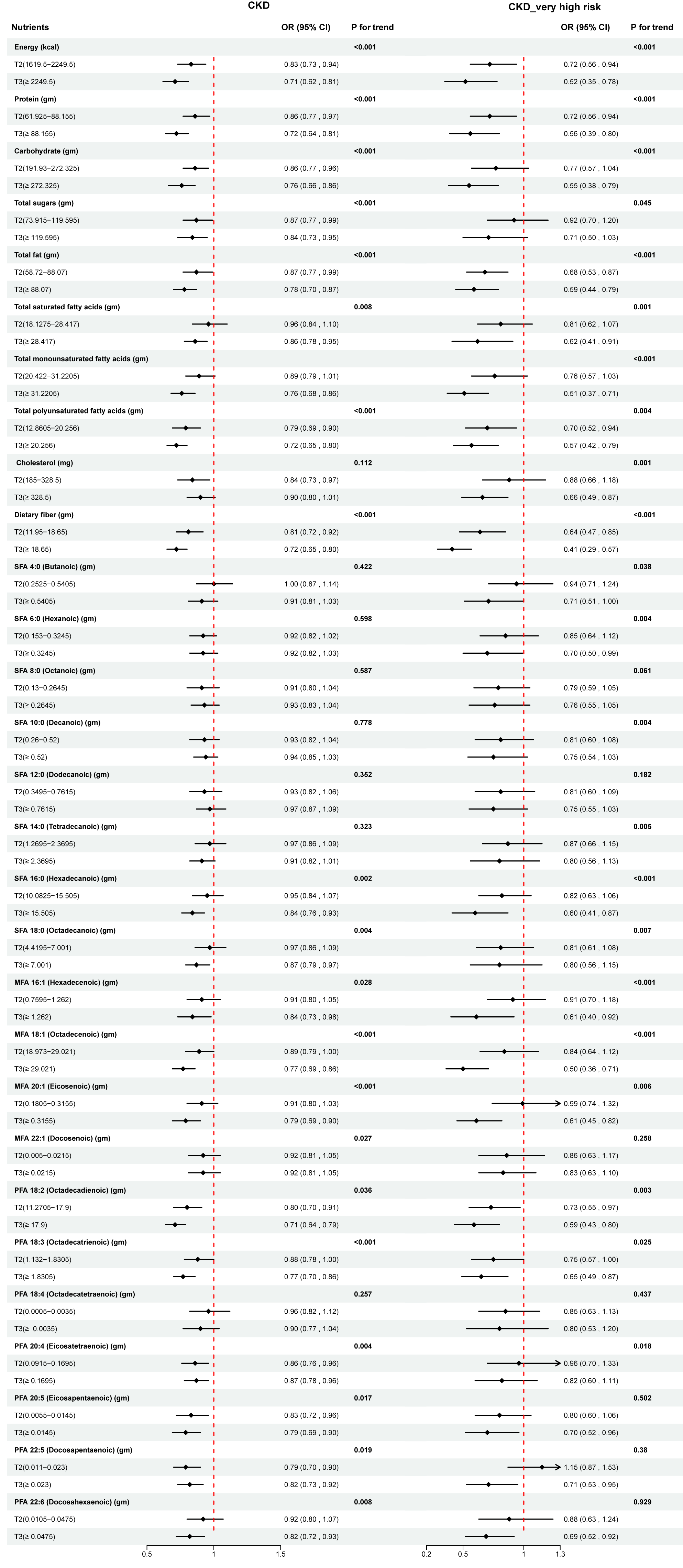

Supplement: Supplementary file 1 [file nutrients-16-02248-s001.zip › Figure S9.tif]
